# Homologous recombination and gene‐specific selection co‐shape the vertical nucleotide diversity of mangrove sediment microbial populations

**DOI:** 10.1002/ece3.70040

**Published:** 2024-07-17

**Authors:** Jijuan Ding, Fei Liu, Jiaxiong Zeng, Hang Gu, Dandan Zhang, Xueqin Yang, Bo Wu, Longfei Shu, Zhili He, Cheng Wang

**Affiliations:** ^1^ School of Environmental Science and Engineering, Southern Marine Science and Engineering Guangdong Laboratory (Zhuhai), State Key Laboratory for Biocontrol Sun Yat‐Sen University Guangzhou China; ^2^ Key Laboratory of Watershed Earth Surface Processes and Ecological Security Zhejiang Normal University Jinhua China

**Keywords:** differentiation, gene‐specific selection, mangrove sediment, microbial population, nucleotide diversity

## Abstract

Mangrove sediments host a diverse array of microbial populations and are characterized by high heterogeneity along their vertical depths. However, the genetic diversity within these populations is largely unknown, hindering our understanding of their adaptive evolution across the sediment depths. To elucidate their genetic diversity, we utilized metagenome sequencing to identify 16 high‐frequency microbial populations comprised of two archaea and 14 bacteria from mangrove sediment cores (0–100 cm, with 10 depths) in Qi'ao Island, China. Our analysis of the genome‐wide genetic variation revealed extensive nucleotide diversity in the microbial populations. The genes involved in the transport and the energy metabolism displayed a high nucleotide diversity (HND; 0.0045–0.0195; an indicator of shared minor alleles with the microbial populations). By tracking the processes of homologous recombination, we found that each microbial population was subjected to different purification selection levels at different depths (44.12% genes). This selection resulted in significant differences in synonymous/non‐synonymous mutation ratio between 0–20 and 20–100 cm layers, indicating the adaptive evolutionary process of microbial populations. Furthermore, our assessment of differentiation in the allele frequencies between these two layers showed that the functional genes involved in the metabolic processes of amino acids or cofactors were highly differential in more than half of them. Together, we showed that the nucleotide diversity of microbial populations was shaped by homologous recombination and gene‐specific selection, finally resulting in the stratified differentiation occurring between 0–20 and 20–100 cm. These results enhance our cognition of the microbial adaptation mechanisms to vertical environmental changes during the sedimentation process of coastal blue carbon ecosystems.

## Graphical abstract

The alleles were widespread but not completely isolated across mangrove sediments. The genes showed wide nucleotide diversity among microbial populations. Gene‐specific selection reduced the non‐synonymous mutants in microbial populations.

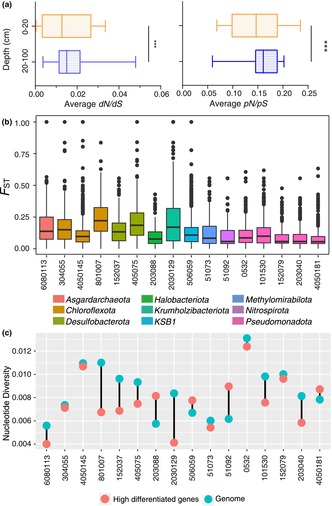

## 1 INTRODUCTION

Mangrove ecosystems were famous for their unique geography and high productivity. Owing to the periodic tides, their environmental conditions varied significantly on a spatiotemporal scale, particularly salinity, nutrient availability, and oxygen concentration, which make these ecosystems a unique biotope. These conditions have contributed to the remarkable microbial diversity that plays essential roles in maintaining the high productivity and ecological processes of mangrove ecosystems. Despite recognition of this substantial functional diversity among the microbial populations in mangrove sediments, their genetic diversity remains largely unexplored. Tracking the genetic diversity and adaptive evolutionary processes of these populations is crucial for comprehending their diversity across highly variable environments.

Nucleotide diversity and differentiation play crucial roles in enabling microbial populations to thrive in changing environments (Hanage, 2016), and evolutionary processes can vary due to natural selection, gene flow, and homologous recombination (González‐Torres et al., 2019). The gene flow would increase the nucleotide diversity of the alleles, which makes it hard to form stable differentiation within the microbial populations. These processes shape microbial evolution and determine whether microbial populations evolve non‐neutrally in different directions to adapt to their surroundings (Polz et al., 2013; Rosen et al., 2015), or if differentiation cannot occur due to sufficient gene flow (Gogarten & Townsend, 2005). Based on these hypotheses, we wonder which evolutionary process would drive the widely distributed microbial populations in the complex and dynamic mangrove sediments. We endeavored to investigate underlying mechanisms driving the genetic diversity and differentiation of microbial populations across the mangrove sediment depths.

Adaptive evolutionary processes of microbial populations were usually distinct across different environments. Previous research has demonstrated that nucleotide diversity and homologous recombination are common within the microbial populations in rivers. However, these populations do not show strong differentiation owing to the presence of ample gene flow (Bouma‐Gregson et al., 2022). Conversely, in marine sediments, nucleotide diversity is consistently low (Starnawski et al., 2017). Compared to these two habitats, the mangrove ecosystems were situated at the interface of river and oceanic environments, experiencing dynamic tidal flooding and draining. Previous studies showed that environmental factors influenced the gene flow of a single species (*Rhizophora racemosa*) in mangrove sediments (Ngeve et al., 2017). However, the genetic diversity at the microbial population level in in‐situ mangrove sediments remains largely unexplored.

We hypothesized that similar to most of the ecosystems, the microbial populations in mangrove sediments would exhibit a wide range of nucleotide diversity. However, deep sediments are less susceptible to environmental fluctuations than surface sediments, which might lead to differences in gene flow and differentiation of microbial populations between different depths. To test our hypotheses, we applied metagenome sequencing to assemble microbial genomes in mangrove sediment cores from Qi'ao Island, a dynamic and representative mangrove ecosystem (Peng et al., 2009). The high‐quality genomes from each microbial populations were selected and compared their adaptive evolutionary processes across the different sediment depths. Our findings showed that a HND (an indicator of shared minor alleles with the microbial populations) and wide homologous recombination were present in more than half of the microbial populations. This study provides a valuable reference for understanding the adaptive evolution and genetic differentiation of microbial populations in mangrove sediments.

## 2 MATERIALS AND METHODS

### 2.1 Sampling and physicochemical properties analyses

We collected five replicates of 100‐cm mangrove sediment cores in December 2019 from Qi'ao Island (22.42° N, 113.63° E) in Zhuhai City, Guangdong Province, China. This location represents a typical natural mangrove ecosystem (Yu et al., 2020) and provides insights into the natural diversity of microbial populations. These five sediment cores were subdivided into 10 depth intervals (0–5, 5–10, 10–15, 15–20, 20–30, 30–40, 40–50, 50–60, 60–80, and 80–100 cm), resulting in a total of 50 sediment samples. After transportation to the laboratory within 24 h using a portable cooler, sub‐samples were stored at −80°C for subsequent DNA extraction, while the remaining samples were utilized for analyses of their physicochemical properties.

We extracted elemental sulfur (ES) using pure methanol (McGuire & Hamers, 2000). The ES content was quantified using high‐performance liquid chromatography equipped with a C18 reverse‐phase column (Eclipse SB‐C18, 5 μm, 150 × 4.6 mm) (Agilent Technologies, Germany) and a UV detector that operated at 254 nm. The salinity was determined using a Pro2Go pH meter (METTLER TOLEDO, Switzerland). The concentrations of sulfite (${\mathrm{SO}}_{3}^{2-}$) and sulfate (${\mathrm{SO}}_{4}^{2-}$) were measured using an iron chromatograph (ICS‐1100; Thermo Fisher Scientific, USA). We analyzed the contents of total carbon, total sulfur, and total nitrogen (TN) using an elemental analyzer (Vario TOC, Elemental, Germany). The CH_4_ content was determined using gas chromatography (7890B GC System; Agilent Technologies, Germany) (Mason et al., 2015). The contents of ferrous ion (Fe^2+^), ferric ion (Fe^3+^), and manganese (Mn^2+^) were extracted by a sequential protocol from a 0.5‐g fresh sample, and these elements were measured using an inductively coupled plasma‐optical emission spectrophotometer (ICP‐OES) (Avio 500; Perkin Elmer, Singapore) (Khan et al., 2022). Ammonium (NH_4_^+^), nitrate (${\mathrm{NO}}_{3}^{-}$), and nitrite (${\mathrm{NO}}_{2}^{-}$) were extracted with 2 M potassium chloride (KCl) from a 2.0‐g fresh sediment and measured using a multimode microplate reader (Varioskan LUX; Thermo Fisher Scientific, USA).

### 2.2 DNA extraction and sequencing

The sediment DNA was extracted with 5.0 g (0–20 cm), 10.0 g (20–60 cm), or 20.0 g (60–100 cm) of mangrove sediments, as previously described (Zhou et al., 1996). Briefly, the mangrove sediment samples were protected by the classic extraction buffer, freeze grind, and sodium dodecyl sulfate lysis. The crude DNA was precipitated using isopropanol at −80°C and purified by Power Soil DNA Isolation Kit (Mo Bio Laboratories, Carlsbad, California, USA). Nanodrop ND‐2000 Spectrophotometer (Thermo Fisher Scientific, MA, USA) was used to determine the DNA purity and concentrations. DNA fragment libraries were subjected to metagenomic sequencing on Illumina Novaseq PE150 at Novogene (Tianjin, China) to generate paired‐end reads (Table S1).

### 2.3 Metagenome sequence assembly, binning, and annotation

We performed quality control using the Read_QC module (default parameters) within the metaWRAP pipeline (v1.3.2) (Uritskiy et al., 2018) with default parameters, yielding clean reads. For higher quality of genomes and deeper coverage, we co‐assembled the five filtered clean read datasets. We applied MEGAHIT (default parameters) for assembling the clean reads and used the binning module (parameters: –maxbin2 –metabat2) within the metaWRAP (Uritskiy et al., 2018) for binning. The quality of the metagenomic‐assembled genomes (MAGs) was estimated by the lineage‐specific workflow of CheckM (v1.0.12) (default parameters) (Parks et al., 2015). CoverM (v0.6.1) was used to calculate the relative abundance of MAGs in the genome mode (parameters: –min‐read‐percent‐identity 0.95 –min‐read‐aligned‐percent 0.75 –trim‐min 0.10 –trim‐max 0.90; https://github.com/wwood/CoverM). The dRep (v3.2.2) (Olm et al., 2017) was used to cluster these MAGs into each populations with 97% average nucleotide identity (ANI) (‐sa 0.97). For each population‐level genomic set, the genome with the high quality was selected as the representative genome (RG). These RGs were assigned to taxonomic classifications using the Genome Database Taxonomy Toolkit (Chaumeil et al., 2019) (GTDB‐Tk, v2.4.0, release 220). The Prodigal (v2.3.6) (Hyatt et al., 2010) was used to predict the open reading frames (ORFs) for these RGs, and these ORFs were subsequently annotated against the Kyoto Encyclopedia of Genes and Genomes (KEGG) database (Kanehisa & Goto, 2000) using kofamScan (v1.3.0) (Aramaki et al., 2020).

### 2.4 Calculation of evolutionary metrics

The population‐level genomic sets, which contained at least five genomes were chosen to track the evolutionary processes. The statistics and evolutionary metrics of the microbial populations, including nucleotide diversity, consensus ANI (conANI), population ANI (popANI), single nucleotide variants (SNVs), linkage disequilibrium (*r* ^2^ and *D*ʹ), non‐synonymous to synonymous mutation ratio (*pN*/*pS*), non‐synonymous to synonymous substitution rate (*dN*/*dS*), coverage, the type of SNVs, and major allele frequency, were calculated from these mappings using the profile module of the inStrain (v1.0.0) program (Olm et al., 2021). For SNP calling, the number of quality‐filtered reads mapping to the position should be at least 5× the coverage and 5% of the SNP frequency, while the reads with the variant base should be above the expected sequencing error rate (1 × 10^−5^) (Dong et al., 2023). We discarded the pairs of SNVs whose distance ≥2.5 SD above the mean (~5%), and the remaining pairs (~95%) were used to calculate their corresponding *r* ^2^ and *D*ʹ. The nucleotide diversity (π) was calculated by summing the squared frequency of each nucleotide across all the positions within a gene using the following equation:

$$\pi =\sum {\left(\mathrm{fA}\right)}^{2}+{\left(\mathrm{fT}\right)}^{2}+{\left(\mathrm{fC}\right)}^{2}+\left(fG^{2}\right)$$

For the pairwise SNVs in a gene, we used the fixation index (*F*_ST_), a measure of the difference in allele frequencies between two microbial populations or subpopulations, to calculate the differentiation between the 0–20 and 20–100 cm by the Hudson method (Bhatia et al., 2013; Hudson et al., 1992) using the following equation:

$$F_{\mathrm{ST}}=\frac{H_{b}-H_{w}}{H_{b}}$$

where *H*_w_ is the mean nucleotide diversity (heterozygosity) within a microbial population and *H*_b_ is the mean nucleotide diversity between two microbial populations.

The Genome Analysis ToolKit (GATK, v4.5.0) (McKenna et al., 2010) and VCFtools (v0.1.16) (Danecek et al., 2011) were used to calculate the *F*_ST_ for the position of each SNV between the 0–20 and 20–100 cm. A custom Python script was used to filter this result (the coverage of each differentia position ≥5).

### 2.5 Statistical analysis

All the statistical analyses were conducted using R (v4.0.5) and Python (v3.7.10), along with the relevant packages. R and Python were used to perform t‐tests (with two‐tailed heteroskedasticity), Wilcoxon tests, Pearson correlations, and linear regressions. The methodology for each statistical analysis is described in conjunction with its results. To reduce dimensionality and analyze the distribution of each MAG within the microbial populations across all the depths, a principal component analysis (PCA) was utilized using the vegan package. The conANI of microbial populations was visualized using ComplexHeatmap (Gu et al., 2016).

## 3 RESULTS

### 3.1 The feature of microbial populations across the mangrove sediment depths

**FIGURE 1:**
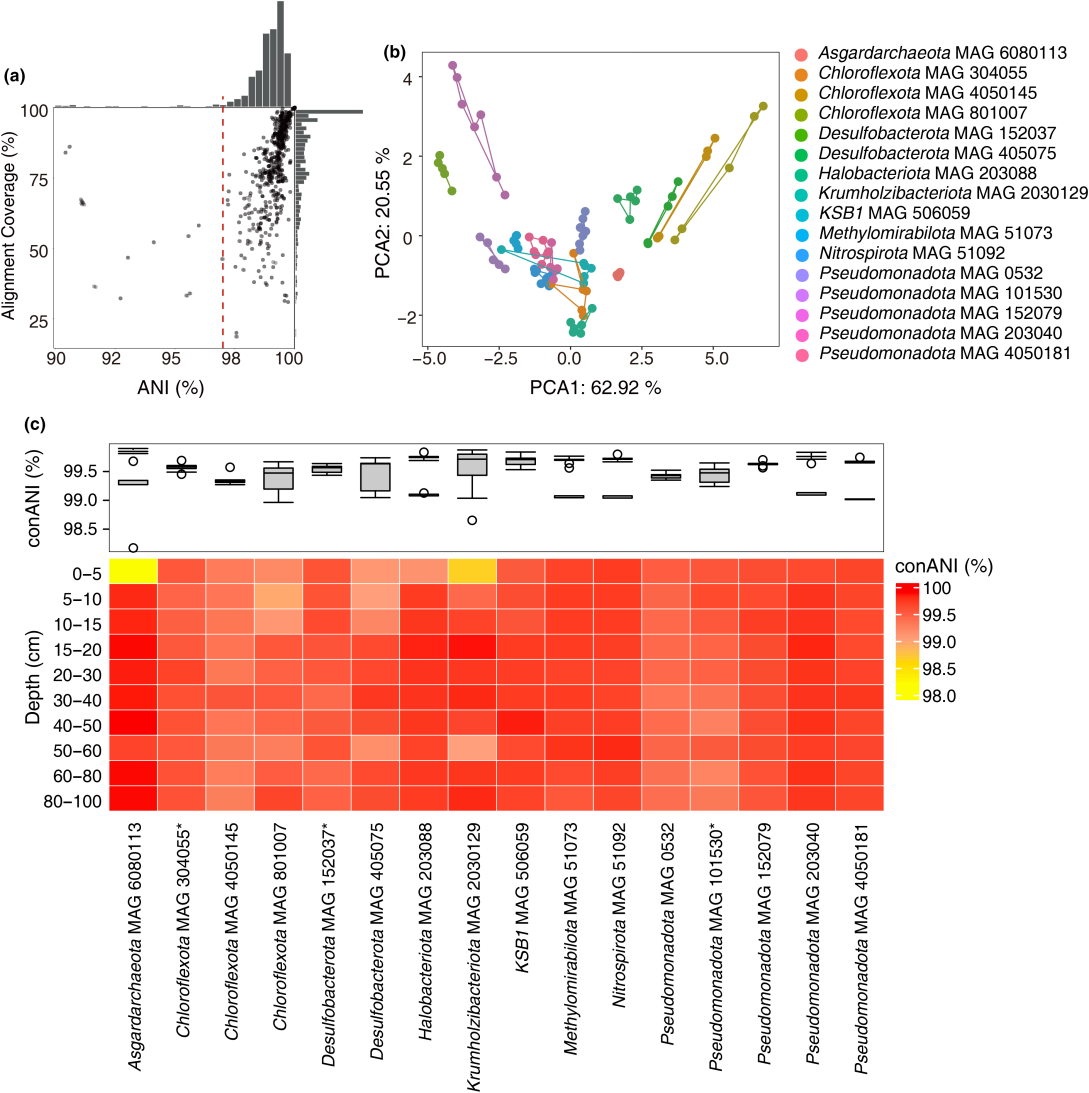
The genomic variability within microbial populations across all the mangrove sediment depths. (a) Histograms of the average nucleotide identity (ANI) and alignment coverage for paired comparisons between all the genomes reassembled from the mangrove sediments. The red line shows the 97% cutoff. (b) PCA plot of the relative abundance of genome within the 16 microbial populations. Each point represents one genome, and the same colors and lines link the same microbial populations. (c) The conANI of the 16 microbial populations across the 10 mangrove sediment depths. The genomes are clustered by Euclidean Distance. The boxplot shows their distributions. * Significant differences of the relative abundance between 0–20 and 20–100 cm (*t*‐test, *p* < .05). conANI, consensusANI; PCA, principal component analysis.

A total of 490 MAGs (completeness ≥50% and contamination ≤10%) were recovered. We calculated the genome‐wide ANI and the alignment coverage for all the pairwise comparisons to determine the optimal parameters for population delimitation (Figure 1a). While the alignment coverage showed a gradual decrease, there was a noticeable drop in the pairwise genome ANI when the ANI cutoff dropped to 97%. This threshold was similar to the one previously reported for defining microbial populations (Crits‐Christoph et al., 2020; Jain et al., 2018). Thus, we used a 97% ANI cutoff to cluster the MAGs into groups of species‐like populations and finally classified 490 MAGs into 228 populations. After classification, 33.47% of the populations could not be assigned to a classified genus, and 95.71% of the populations could not be assigned to a species (Figure S1). This suggests the presence of many uncultured or even undiscovered microbial populations in 100‐cm mangrove sediment columns.

Among these populations, we selected high‐frequency populations with at least five MAGs that were individually assembled from different sediment depths, resulting in a set of 16 high‐frequency populations (14 bacterial and two archaeal populations) (Table S2) with an ANI that ranged from 98.15% to 99.84%. Here, high‐frequency species were targeted since they were more likely to have adaptive evolutionary processes with the change of sediment depth. These populations were affiliated with various groups commonly found in mangrove sediments, including *Chloroflexota*, *Pseudomonadota*, *Desulfobacterota*, *Methylomirabilota*, *Asgardarchaeota*, and *Nitrospirota* (Andreote et al., 2012; Lin et al., 2019; Wang et al., 2022). The *Chloroflexota*, *Pseudomonadota*, and *Desulfobacterota* constitute the dominant parts of microbial communities in mangrove sediments (Zhang, Liu, Pan, et al., 2023). The *Methylomirabilota* and *Asgardarchaeota* were closely related to the organic carbon degradation process of mangrove sediments (Cai et al., 2021), while *Nitrospirota* links anaerobic methane oxidation to nitrite denitrification through a unique, O_2_‐producing, internally aerobic methanotrophic pathway in mangrove sediments (He et al., 2015). Other populations, such as *Krumholzibacteriota* and *Candidatus KSB1*, were infrequently reported in previous studies (Zhang, Liu, Cha, et al., 2023). This would provide insight into the deeper‐varying genetic diversity of these specific and widely reported microorganisms in mangrove sediments. The PCA analyses revealed that the in‐depth abundance profiles of MAGs within each microbial population were highly similar (Figure 1b). Therefore, we selected one RG for each of the 16 populations based on the highest completeness and lowest contamination. The relative abundance of these selected populations ranged from 0.002% to 0.072% across the 10 sediment depths (Figure S2). To further investigate the difference within these 16 microbial populations, we compared the conANI (the ANI of the dominant genotype) across the 10 depths (Figure 1c). Data revealed that the conANI of 16 microbial populations ranged from 98.17% to 99.90% and varied with depths, but only three of them showed a significant difference between the 0–20 and 20–100 cm (*t*‐test, *p* < .05), including *Chloroflexota* MAG 304055, *Desulfobacterota* MAG 152037, and *Pseudomonadota* MAG 101530. ConANI's inconsistency suggests that the percentage of major allele frequency within microbial populations was distinctive among different depths. In addition, the popANI (a measure that includes consideration of the variants in subdominant genomes and minor alleles) ranged from 98.69% to 99.99% (Figure S3), and nine of them differed significantly between the 0–20 and 20–100 cm (*t*‐test, *p* < .05). The variation of popANI (a measure of variants) was lower than that of conANI (dominant genotype), thus the evolutionary process of these microbial populations was not a random direction, but a selective consensus. This result indicates that the long‐term selective evolution (conANI) was the dominant force rather than random mutation (popANI) in these microbial populations across mangrove sediments, and the alleles within these microbial populations were widespread but not completely isolated.

### 3.2 Genomic nucleotide diversity within the microbial populations

**FIGURE 2:**
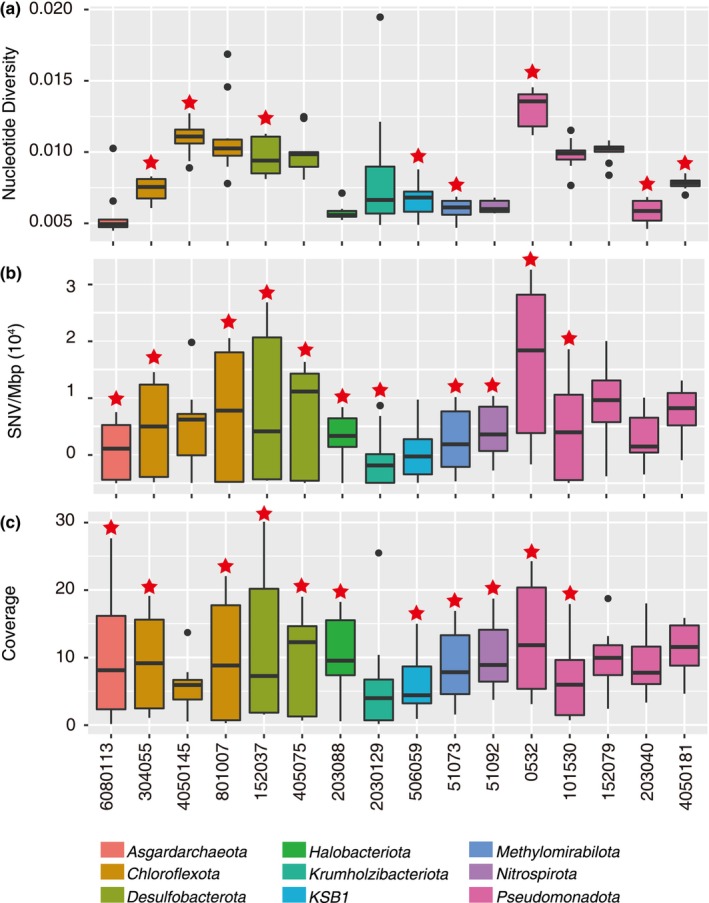
Genetic diversity within the microbial populations across all the mangrove sediment depths. The boxplot shows the microbial nucleotide diversity (a), SNV/Mbp (b), and coverage (c) across the 10 mangrove sediment depths. The line in the box represents the average, and the dots are outliers. The box color shows the classification at the phylum level. The red start above the box shows the significant differences between 0–20 and 20–100 cm (*t*‐test, *p* < .05). SNV, single nucleotide variant.

To further investigate the genomic variability within the 16 microbial populations, we calculated their nucleotide diversity, SNV per megabase (SNV/Mbp), and coverage across all the depths. Here, nucleotide diversity is a measurement of genetic diversity in a population, SNV/Mbp is a measure of the number of SNV sites per average 1Mbp, and coverage is used to show the distribution of microbial populations in different samples. Our findings showed that the average nucleotide diversity ranged from 0.0045 to 0.0195 (Figure 2a, Table S3); the average SNV/Mbp ranged from 2 to 37,558 (Figure 2b), and the average coverage ranged from 0.14 to 30.09 (Figure 2c) across the 16 microbial populations. The *k*‐mean clustering method was used to cluster the indexes of microbial populations, including nucleotide diversity, SNV/Mbp and coverage (Figure S4), and we found significant genomic differences between the 0–20 and 20–100 cm (*t*‐test, *p* < .05).

To assess the best indicator of genetic diversity within the microbial populations, we compared the relationships between nucleotide diversity, SNV/Mbp, and coverage. We observed a weak correlation between coverage and nucleotide diversity or SNV/Mbp (Figure S5, linear regression). In contrast, there was a strong correlation of the nucleotide diversity with the SNV/Mbp (Figure S5, linear regression, *R* ^2^ = .42, *p* ≤ .05). As the depth and coverage are interchangeable, our results suggest that the nucleotide diversity and SNV/Mbp are intrinsic characteristics of the microbial populations and are not significantly impacted by the sequencing process. Thus, in this study, we identified nucleotide diversity as a representative feature of genetic mutations within the microbial populations.

**FIGURE 3:**
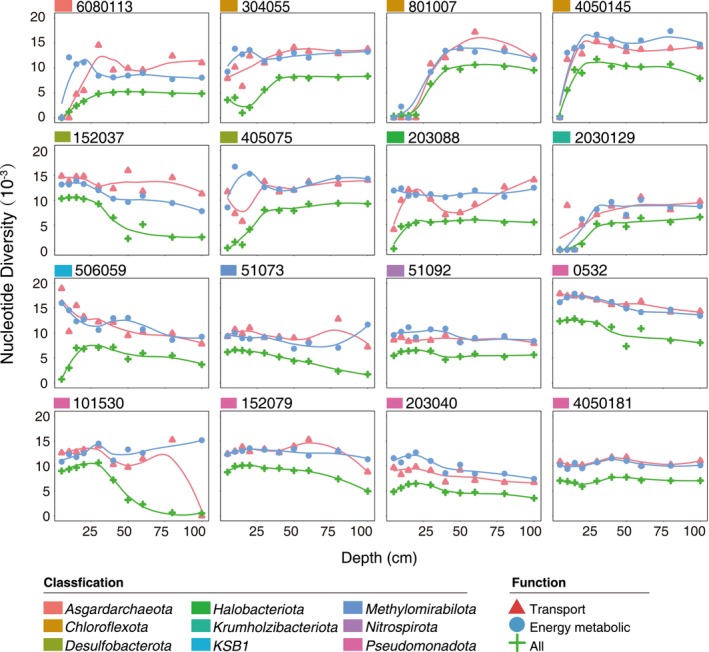
The distributions of nucleotide diversity of selected high nucleotide diversity (HND) genes within microbial populations across all the mangrove sediment depths. Average nucleotide diversity of selected HND genes within the microbial populations across the 10 depths. The color is separated by transport genes (red), energy metabolic genes (blue), and all genes (green). The curves are fitted to their trends across the 10 depths. The box color shows the classification at the phylum level.

We further screened out HND genes with values ≥2.5 SD above the mean in each of the 16 microbial populations across the 10 depths, resulting in a range of 279–1465 HND genes. After functional annotation against the KEGG database (Kanehisa & Goto, 2000), we found that these HND genes were involved in nine to 45 metabolic pathways (Figure S6). Among them, only the HND genes involved in transport (e.g., transporters, exosomes, and secretion systems) were detected within all the microbial populations across the 10 depths (Figure 3). These HND genes were primarily involved in the processes of signaling and cellular processes, membrane transport, and carbohydrate metabolism. With the exception of *Nitrospirota* MAG 51092 and *Halobacteriota* MAG 203088, the most frequent HND genes (10 of 95 genes) of nearly all the microbial populations were involved in transport, suggesting the frequent signal communication and metabolic exchanges in the microbial populations from the mangrove sediments (Table S4). In addition, except for *Pseudomonadota* MAG 0532, the HND genes involved in the energy metabolic process were also enriched in all the depths (Figure 3). Interestingly, unlike the HND genes involved in many different energy metabolic pathways, such as oxidative phosphorylation, nitrogen metabolism, or sulfur metabolism, in the bacterial populations, the HND genes involved in the energy metabolic pathways in the archaeal populations (*Asgardarchaeota* MAG 6080113 and *Halobacteriota* MAG 0203088) were primarily concentrated in methane metabolism. These findings suggest that the HND genes, that were closely related to the energy metabolism, showed a population‐wide nucleotide diversity in mangrove sediments. The diversity of energy metabolism genes in microbial populations is the basis of the functional diversity of microbial communities in mangrove sediments (Padhy et al., 2022; Zhang et al., 2021).

To investigate whether the genetic variation of microbial populations in mangrove sediments was influenced by environmental variables, we analyzed the correlation between these 16 microbial populations and 14 environmental factors (Table S5). The result revealed that the impact of environmental factors on different populations was inconsistent (Figure S7). As the most influential factors, NH_4_^+^ and TN significantly impacted the nucleotide diversity of seven populations. Our results suggest that changes in nitrogen have an important effect on the nucleotide diversity of microbial communities at the vertical scale. They negatively impacted *Pseudomonadota* MAG 4050181 and *Chloroflexota* MAG 304055 but positively impacted *Pseudomonadota* MAG 203040, *Pseudomonadota* MAG 0532, *Methylomirabilota* MAG 51073, *Desulfobacterota* MAG 405075, and *Desulfobacterota* MAG 152037.

### 3.3 Homologous recombination within the microbial populations

**FIGURE 4:**
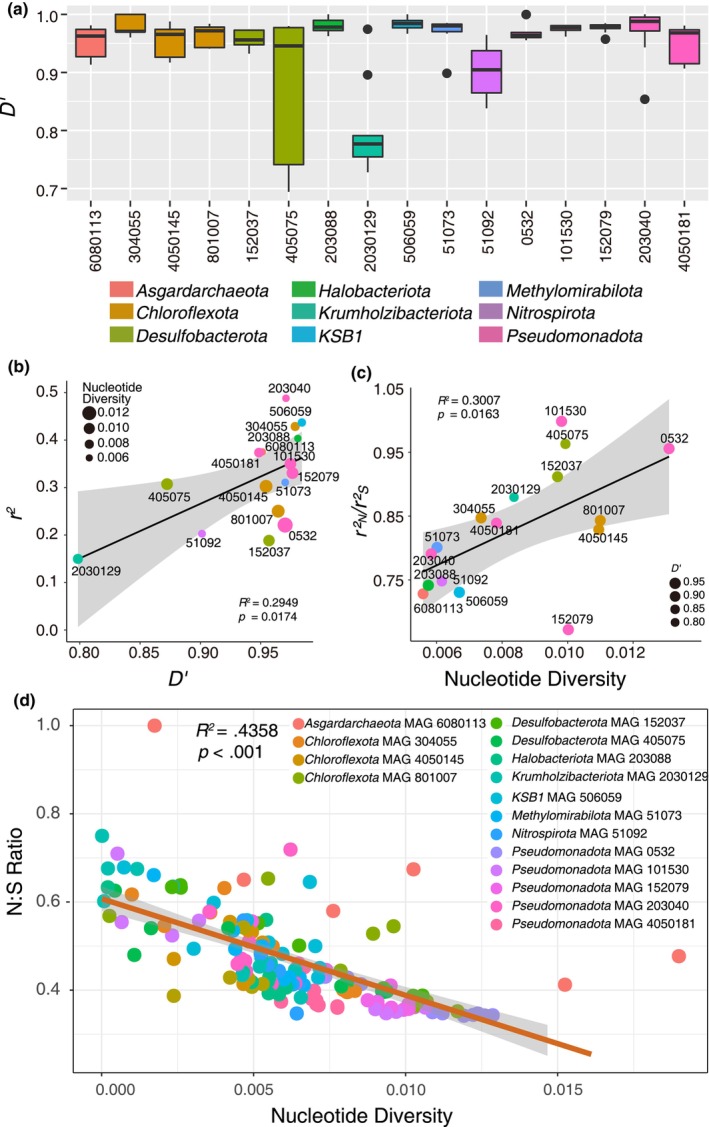
The homologous recombination and stratification within the microbial populations. (a) The boxplot shows *D*ʹ within 16 microbial populations across all the depths. The line in the box represents the average, and the dots are outliers. The box color shows the classification at the phylum level. (b) The relationship between average *r* ^2^ and the average *D*ʹ within the 16 microbial populations. A linear regression model (*R* ^2^ = .3898, *p* = .0033) is shown. The size of each point represents the average microbial nucleotide diversity. (c) The relationship between average nucleotide diversity and the average ratio of linkage of non‐synonymous–non‐synonymous versus synonymous–synonymous ($r_{N}^{2}/r_{S}^{2}$) paired SNVs. A linear regression model (*R* ^2^ = .2681, *p* = .027) is shown. The size of each point represents the average *D*ʹ value within the microbial population. (d) The relationship between nucleotide diversity and the non‐synonymous/synonymous (N:S) SNVs ratio within 16 microbial populations. Each point represents the nucleotide diversity observed within each microbial population at each depth. A linear regression (*R* ^2^ = .4348, *p* < .001) is shown. SNVs, single nucleotide variants.

To examine whether the HND within the 16 selected microbial populations could be inherited, we investigated the occurrence of homologous recombination within each microbial population in all the depths. Using the *r* ^*2*^ and *D*ʹ values between two SNVs within the scaffold as the indicators of linkage disequilibrium (VanLiere & Rosenberg, 2008), we found that the mean distance between the two SNVs was 34 bp (Table S3). When the distance was ≥2.5 SD above the mean, their corresponding *r* ^2^ and *D*ʹ were unreliable. As expected, the *r* ^*2*^ of linkage disequilibrium decay as the genomic distance between the two SNV sites increased across all 16 microbial populations (Figure S8), indicating the widespread homologous recombination within a population and the foundation for their adaptive evolution. To further confirm the existence and differences of homologous recombination across the microbial populations, we compared the average *D*ʹ, an alternative metric of linkage disequilibrium. In the 16 microbial populations, the average *D*ʹ ranged from 0.7985 to 0.9841 (Figure 4a), which also indicated the occurrence of linkage disequilibrium in all the microbial populations. However, it was not true that all the pairwise SNVs showed linkage disequilibrium because we found *D*ʹ < 1 occurring at a significant fraction (~44.12%) of each paired site (Figure S9). This indicates the occurrence of non‐neutral homologous recombination in all the depths. A positive correlation was also observed between the average *D*ʹ and mean *r* ^*2*^ (Figure 4b, linear regression; *R* ^2^ = .29; *p* = .017) within the microbial populations. These results suggest that homologous recombination coexists with linkage disequilibrium in these 16 microbial populations, supporting the ongoing processes of non‐neutral homologous recombination within the microbial populations.

To further verify how gene selections result in non‐neutral homologous recombination, we performed the related markers for synonymous or non‐synonymous SNVs within 16 microbial populations across all the depths. In this study, the ratio of *r* ^2^_N_/*r* ^2^_S_ increased with the nucleotide diversity (Figure 4c, linear regression; *R* ^2^ = .3007; *p* = .0163), suggesting that the ratio of beneficial or slightly deleterious non‐synonymous SNVs increased with higher nucleotide diversity. Except *Pseudomonadota* MAG 101530, nearly all the microbial populations had an *r* ^2^_N_/*r* ^2^_S_ ≤ 1. Such a lower degree of coupling linkage for non‐synonymous variants indicates that the inheritance of non‐synonymous–non‐synonymous SNVs was difficult during the process of recombination. We further enumerated the non‐synonymous/synonymous ratio (N/S ratio) and its associations with nucleotide diversity. Notably, all the microbial populations except *Asgardarchaeota* MAG 6080113 at the 0–5 cm had an N/S ratio ≤ 1 (Figure 4d, linear regression, *R* ^2^ = .4358, *p* < .001), indicating that the purifying selection accumulated more synonymous SNVs than non‐synonymous ones. In addition, we found no relationship between the nucleotide diversity and the microbial population abundance (Figure S10, linear regression; *p* = .9363). These results suggest that gene selection contributes to the reduction in the non‐synonymous SNVs in these 16 microbial populations.

### 3.4 Highly differentiated genes within microbial populations

**FIGURE 5:**
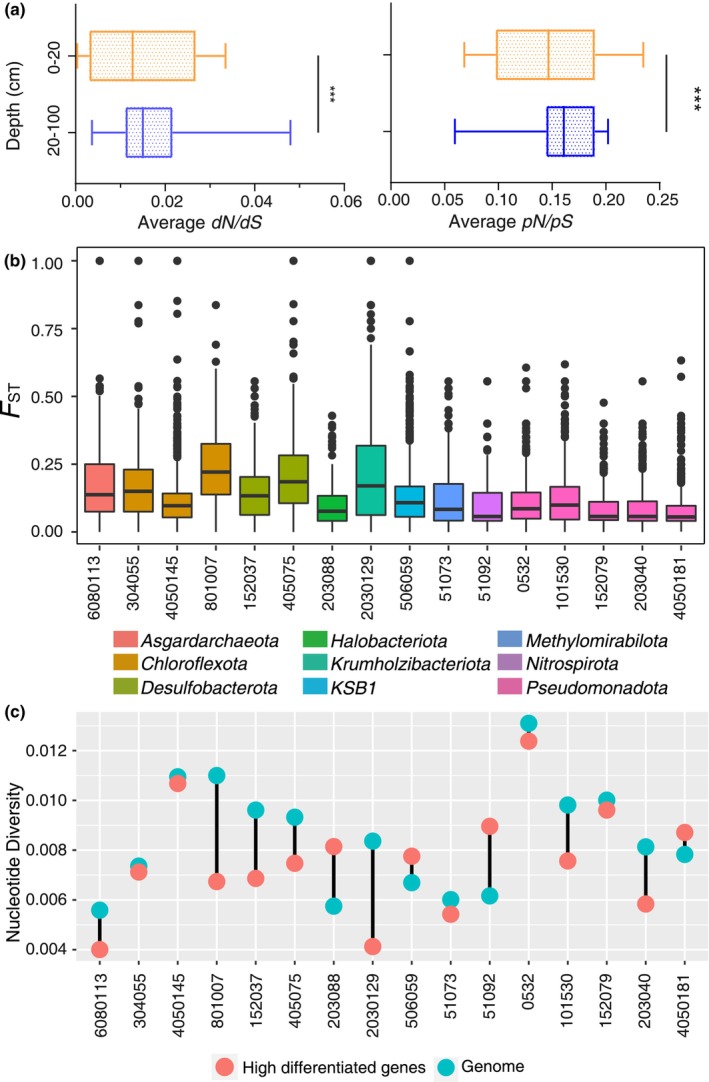
The degree of differentiation and the highly differentiated genes within each microbial population. (a) Average *pN*/*pS* and *dN*/*dS* of the 16 microbial populations between 0–20 and 20–100 cm. The short line within the box shows the average. *** *p* < .001 (*t*‐test). (b) The boxplot shows the *F*_*ST*_ value between the 0–20 cm and 20–100 cm within each microbial population and the color represents their classification at the phylum level. The points show the abnormal genes. (c) The comparison of average nucleotide diversity between the highly differentiated genes (red circle) and the average genes (blue circle) within each microbial population.

The presence of non‐neutral homologous recombination within microbial populations led us to explore whether the microbial populations were stratified and differentiated. To determine this, we compared the ratio of non‐synonymous SNVs to synonymous SNVs and the substitution rate between 0–20 and 20–100 cm. Data revealed that the average *pN*/*pS* and *dN*/*dS* were ≤1 within all the microbial populations across all the depths, which confirmed that the gene selection could reduce the ratio and substitution rate of non‐synonymous SNVs in all the depths. Moreover, the average of *pN*/*pS* and *dN*/*dS* at 0–20 cm was significantly smaller than that at 20–100 cm (Figure 5a, Wilcoxon test, *p* < .05). To support it, we also compared the allele frequency within the microbial populations between 0–20 and 20–100 cm. Our findings showed that the average major allele frequency at 0–20 cm (0.6368) was significantly higher than that at 20–100 cm (0.6311) when the *D*ʹ was ≤1 between the two SNVs (*t*‐test, *p* = .0038). These results suggest that these microbial populations might be subject to a more complex selection at 0–20 cm compared to that at 20–100 cm.

Based on these results, we compared the difference in SNV number, nucleotide diversity, and *D*ʹ within the microbial population between 0–20 and 20–100 cm. Our results revealed that 11 microbial populations had a significant difference in their SNV number (*t*‐test, *p* < .05), and eight microbial populations also had a significant difference in nucleotide diversity (*t*‐test, *p* < .05). Conversely, only three microbial populations differed significantly in their major allele frequency (MAF) (*t*‐test, *p* < .05), and only five microbial populations showed a significantly different *D*ʹ between 0–20 and 20–100 cm (*t*‐test, *p* < .05). Considering that the MAF and *D*ʹ are the important indicators for assessing the genetic heterogeneity of microbial populations (Dong et al., 2023), these results indicate that genetic heterogeneity occurred within over half of microbial populations, but fewer mutants were retained during genetic selection.

Since our correlation analysis found the inconsistent effects of environmental factors on microbial populations (Figure S6), we further endeavored to identify the genes that underwent gene‐specific selection, known as highly differentiated (HD) genes. To do so, we calculated the pairwise fixation index (*F*_ST_) per gene between 0–20 and 20–100 cm. When the specific loci show higher *F*_ST_ than the average *F*_ST_ of the genome, we could assume that this locus experienced population‐specific selective pressures (Bouma‐Gregson et al., 2022; Crits‐Christoph et al., 2020). Data revealed that the *F*_*ST*_ ranged from 0.0078 to 0.2358 within all the microbial populations (Figure 5b). We then screened out 378 HD genes with an *F*_ST_ ≥ 2.5 SD above the genomic mean (Table S6) and observed that these HD genes had a lower nucleotide diversity compared with their genomes, with the exception of *Pseudomonadota* MAG 4050181, *Nitrospirota* MAG 51092, *KSB1* MAG 506059, and *Halobacteriota* MAG 203088 (Figure 5c). This may be owing to the removal of deleterious non‐synonymous mutations by purifying selection, which enabled beneficial mutations to genetically differentiate through homologous recombination between the 0–20 and 20–100 cm (Hughes, 2005). The microbial populations varied in their number of HD genes, with the most found in *Methylomirabilota* MAG 51073 (39) and the least found in *Asgardarchaeota* MAG 6080113 and *Chloroflexota* MAG 801007 (10). After functional annotation, we found that 13 microbial populations contained HD genes involved in amino acid metabolism; 10 contained genes related to transport, and 8 contained genes involved in the metabolism of cofactors, primarily vitamins. These findings suggest that gene‐specific selections occur between 0–20 and 20–100 cm in the mangrove environments.

## 4 DISCUSSION

In this study, we examined 16 high‐frequency microbial populations from 0 to 100 cm mangrove sediments. Our analysis of their nucleotide diversity and homologous recombination events revealed a varying pattern of genetic diversity between 0–20 and 20–100 cm. In addition, we observed a wide range of homologous recombination and genetic differentiation among each microbial population across the different sediment depths. This was particularly true for the genes involved in the metabolic processes for amino acids and cofactors. These findings provide a valuable reference for exploring the adaptive evolutionary processes of microbial populations in highly dynamic habitats.

Our findings indicate that the energy metabolism exhibits HND within the microbial populations across different depths of mangrove sediments, which was accompanied by the change in environmental factors across different depths. Considering the significance of nitrogen metabolism as an essential energy metabolic pathway in the microbial community of mangrove sediments (Bai et al., 2013), we observed that the nitrogen elements (TN and NH_4_^+^) exerted a notable influence on seven microbial populations. These influences were determined by the distinct energy metabolism characteristics exhibited by these microbial populations. For example, *Pseudomonadota* MAG 203040 and *Pseudomonadota* MAG 0532 were associated with the oxidation of NH_4_^+^ (Hayatsu et al., 2017), whereas *Methylomirabilota* MAG 51073 utilized nitrogen elements to facilitate the anaerobic oxidation of methane (Wallenius et al., 2021). Consequently, the decrease in nucleotide diversity correlated with the decreasing levels of TN and NH_4_^+^. However, the nitrogen cycle in mangrove sediments displayed remarkable plasticity and is interrelated with other energy metabolic activities, such as methane and sulfur metabolism (Li et al., 2018; Wallenius et al., 2021). This suggests that alterations in the nitrogen level directly impact the nucleotide diversity of energy metabolic pathways in various microbial populations. Since environmental factors played a decisive role in the ecological functioning of microorganisms (Wang et al., 2019), our results suggest that the nucleotide diversity of microbial populations has the potential to indicate the environmental conditions in mangrove sediments.

Our correlation analysis showed that the nucleotide diversity of 16 populations was influenced by complex environmental factors. Since the nucleotide diversity is not necessarily inherited in microbial populations (de Vries et al., 2020), we hypothesized that the different effects from environmental factors may lead to the gene‐specific selection, subsequently resulting in non‐neutral inheritance in the microbial populations during the processes of homologous recombination. Therefore, tracing HD functional genes within microbial populations is crucial for understanding their adaptive evolution. Most of the microbial populations significantly differed in gene heterogeneity between the 0–20 and 20–100 cm. However, such differences were not observed in the occurrence of homologous recombination events between the 0–20 and 20–100 cm, suggesting the presence of depth‐dependent non‐neutral genetic selection. The more extensive genetic selections at the 0–20 cm than at the 20–100 cm indicated that the more complex environmental changes occurred at the depth of 0–20 cm. This might be one key reason why more non‐synonymous SNVs were erased at 0–20 cm than at 20–100 cm.

By calculating the *F*_ST_, our study tracked the differentiation degree of genes between the 0–20 and 20–100 cm. The HD genes were identified as related to the metabolic processes of amino acids or cofactors. These amino acids and cofactors, are essential substances for microbial life (Gupta et al., 2020) and can mediate signaling processes among the microbial populations (Lawson et al., 2017; Sokolovskaya et al., 2020). These HD genes were detected in more than half of the microbial populations, and their coverage was consistent with the variability within the microbial populations. This data supports the importance of the genes related to amino acids and cofactors during the process of microbial adaption and evolution (Mayr et al., 2021; Rocha & Danchin, 2004). Furthermore, these HD genes had a lower nucleotide diversity and *pN*/*pS* than the genomic average, which were the signals for the purifying selections (Crits‐Christoph et al., 2020). As noted earlier, changes in the nitrogen and sulfur elements were significantly related to nucleotide diversity. These elements not only provide energy for microbes but are also essential elements in the composition of amino acids and cofactors. Together, the vertical differentiation of these genes was shaped by homologous recombination and gene‐specific selection in mangrove sediments.

## 5 CONCLUSIONS

We discovered that the 16 selected microbial populations displayed widespread nucleotide diversity across 100 cm mangrove sediment depths. The genes with the highest nucleotide diversity were found to be associated with transport and energy metabolism functions. Furthermore, our data showed that homologous recombination and depth‐dependent gene‐specific selection took place differently between 0–20 and 20–100 cm, which led to a high degree of differentiation in the genes involved in amino acids and cofactors. These findings suggest that environmental changes play a role in shaping the nucleotide diversity and differentiation of the microbial populations, leading to their adaptive evolution across the sediment depths. This study advances our understanding of the genetic variation of microbial populations in mangrove sediments. However, the limitations in the sequencing depth and the low cultivability of microbes (Stewart, 2012) suggest that more studies are needed to comprehensively investigate the microbial adaptive evolution mechanism and the relationships between the evolutionary processes and environmental factors across mangrove sediment columns.

## AUTHOR CONTRIBUTIONS

**Jijuan Ding:** Conceptualization (equal); data curation (equal); formal analysis (equal); investigation (equal); methodology (equal); resources (equal); software (equal); validation (equal); visualization (equal); writing – original draft (equal); writing – review and editing (equal). **Fei Liu:** Conceptualization (equal); data curation (equal); formal analysis (equal); investigation (equal); methodology (equal); software (equal); writing – review and editing (equal). **Jiaxiong Zeng:** Data curation (equal); formal analysis (equal); investigation (equal); resources (equal); software (equal). **Hang Gu:** Formal analysis (equal); investigation (equal); resources (equal). **Dandan Zhang:** Data curation (equal); formal analysis (equal); investigation (equal); resources (equal); visualization (equal). **Xueqin Yang:** Data curation (equal); formal analysis (equal); validation (equal). **Bo Wu:** Data curation (equal); formal analysis (equal); resources (equal). **Longfei Shu:** Project administration (equal); resources (equal); writing – original draft (equal); writing – review and editing (equal). **Zhili He:** Conceptualization (equal); funding acquisition (equal); project administration (equal); resources (equal); writing – original draft (equal); writing – review and editing (equal). **Cheng Wang:** Data curation (equal); formal analysis (equal); funding acquisition (equal); project administration (equal); software (equal); supervision (equal); writing – original draft (equal); writing – review and editing (equal).

## FUNDING INFORMATION

This study was supported by the National Natural Science Foundation of China (92251306, 52070196, 32370113, 32300109), the Guangdong Basic and Applied Basic Research Foundation (2024A1515010931), and the Southern Marine Science and Engineering Guangdong Laboratory (Zhuhai) (SML2023SP218, SML2020SP004).

## CONFLICT OF INTEREST STATEMENT

The authors declare that they have no competing interests.

## Supporting information


Figure S1

## Data Availability

The raw reads of metagenomes were submitted to the National Center for Biotechnology Information Short Reads Archive (NCBI SRA) database under the project PRJNA798446 and the National Omics Data Encyclopedia (NODE) under the project OEX012906. All of the codes and metrics are available at https://github.com/FeiLiu17/liufei.git.
