# Supplementary material for: Homologous recombination and gene‐specific selection co‐shape the vertical nucleotide diversity of mangrove sediment microbial populations
Source: Ecol Evol. 2024 Jul 17;14(7):e70040. doi: 10.1002/ece3.70040 (PMC11254452; doi:10.1002/ece3.70040)
Supplement: Supplementary file 1 — Figure S1 [file ECE3-14-e70040-s001.docx]

**Supplementary information**

**Homologous recombination and** **gene-specific selection co-shape the vertical nucleotide diversity of mangrove sediment microbial populations**

Jijuan Ding^1a^, Fei Liu^1a^, Jiaxiong Zeng^1^, Hang Gu^1^, Dandan Zhang^1^, Xueqin Yang^1^, Bo Wu^1^, Longfei Shu^1^, Zhili He^1*^, Cheng Wang^1, 2*^

^1^School of Environmental Science and Engineering, Southern Marine Science and Engineering Guangdong Laboratory (Zhuhai), State Key Laboratory for Biocontrol, Sun Yat-sen University, Guangzhou 510006, China

^2^Key Laboratory of Watershed Earth Surface Processes and Ecological Security, Zhejiang Normal University, Jinhua 321004, China

*** Corresponding authors:** Cheng Wang ([wangcheng5@mail.sysu.edu.cn](mailto:wangcheng5@mail.sysu.edu.cn)), or Zhili He ([hezhili@sml-zhuhai.cn](mailto:hezhili@sml-zhuhai.cn)).

## Figure and Table legends

**Fig. S1** Total genomes unclassified at each taxonomic level. The genomes are dereplicated at the population level (i.e., 97% ANI). ANI, average nucleotide identity.

**Fig. S2** Relative abundance of genomes within the 16 microbial populations across all the depths. The heatmap of DNA relative abundance within the 16 microbial populations across the mangrove sediment depths and clustered by *Euclidean Distance.* The boxplot shows their distributions. **p* < 0.05 for the relative abundance between 0-20 cm and 20-100 cm (*t*-test).

**Fig. S3** Population ANI (popANI) within the 16 microbial populations across all the depths. The heatmap of popANI within the 16 microbial populations across all the depths and clustered by Euclidean Distance*.* The boxplot shows their distributions.

**Fig. S4** The tree plots to the right of these boxplots cluster the features of the microbial community at different depths. The tree was clustered by ward.D2.

**Fig. S5** The relationships between coverage, nucleotide diversity, and the SNV/Mbp. The relationship between coverage with SNVs/Mbp (linear regression, *R^2^* = 0.0353), nucleotide diversity with the SNVs/Mbp (linear regression, *R^2^* = 0.4219), and nucleotide diversity with coverage (linear regression, *R^2^* < 0.001). Each point represents one gene. SNVs, single nucleotide variants.

**Fig. S6** Annotation of the high nucleotide diversity genes within the 16 microbial populations. The genes whose nucleotide diversity > 2.5 SDs above the average were selected and annotated against the KEGG database. KEGG, Kyoto Encyclopedia of Genes and Genomes.

**Fig. S7** The relationships between the 16 microbial populations and environmental factors. The colors represent the positive (red) or negative (blue) relationships. The size of the circle represents the strength of the correlation. The circle is only displayed when *p* < 0.05.

**Fig. S8** Linkage disequilibrium decay over genomic distance within the 16 microbial populations. Each point represents a mean of linkage for the SNVs at that genomic distance. They are divided into nonsynonymous-nonsynonymous linkages, nonsynonymous-synonymous linkages, and synonymous-synonymous linkages. The size of each point represents the number of SNVs that went into calculating the average. SNVs, single nucleotide variants.

**Fig. S9** The percentage of genes with an average *D'* < 1. Each box represents one microbial population. The colors represent the phylum, and the short line within the box shows the average. Points above the whiskers indicate outliers.

**Fig. S10** Relationships between nucleotide diversity and DNA relative abundance. The relationship between the average nucleotide diversity and relative abundances within the microbial populations across all the depths.

**Table S1** The total base and reads number of each clean data.

**Table S2** Completeness, contamination, and classification for each reference genome within the 16 microbial populations.

**Table S3** Summary of key population genetics statistics.

**Table S4** List of high nucleotide diversity genes. Only the high nucleotide diversity genes obtained within more than eight microbial populations are shown in the table.

**Table S5** The environmental factors in 0-100 cm mangrove sediments.

**Table S6** Annotations and the key genetic statistics of the highly differentiated genes.


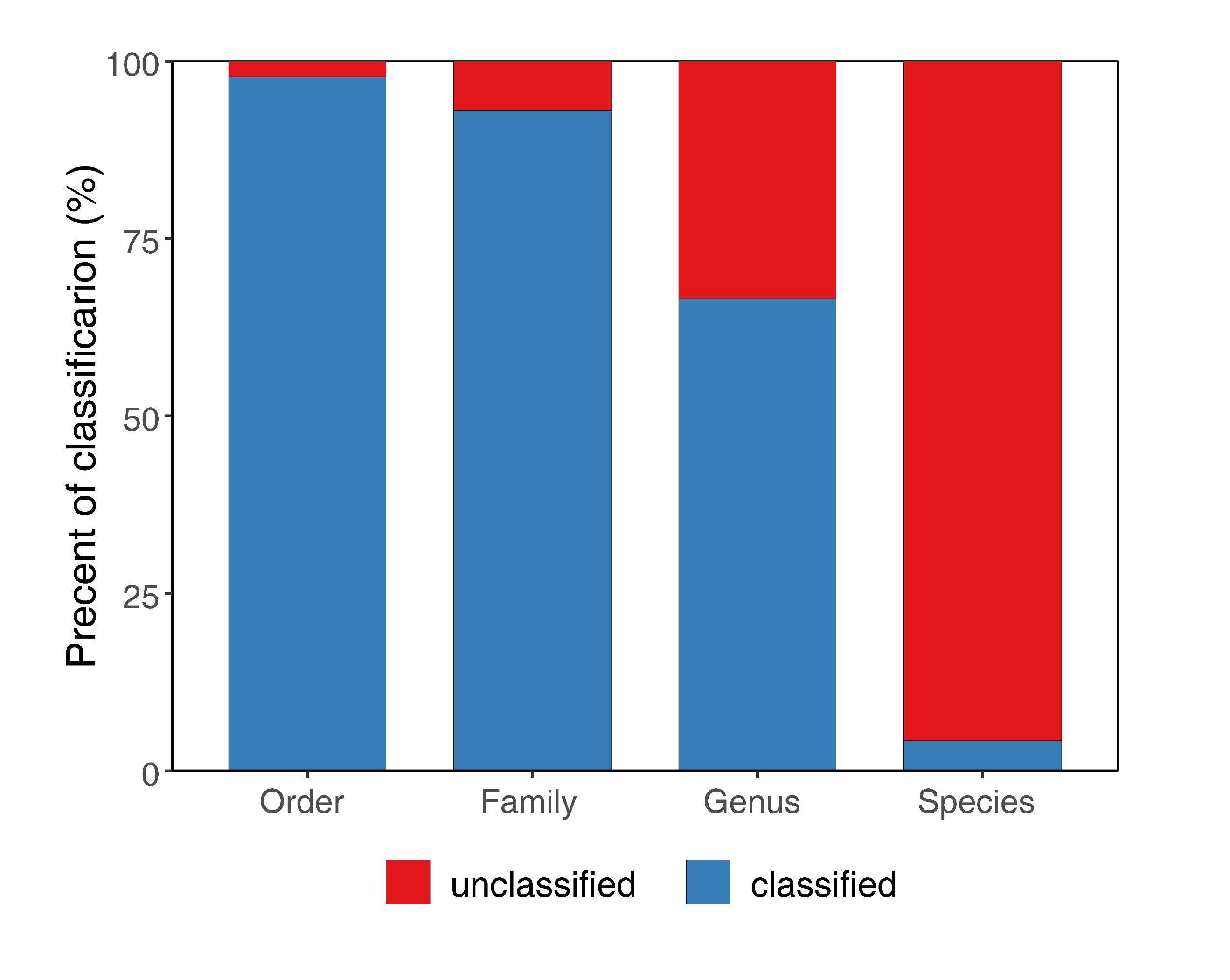


**Fig. S1** Total genomes unclassified at each taxonomic level. The genomes are dereplicated at the population level (i.e., 97% ANI). ANI, average nucleotide identity.


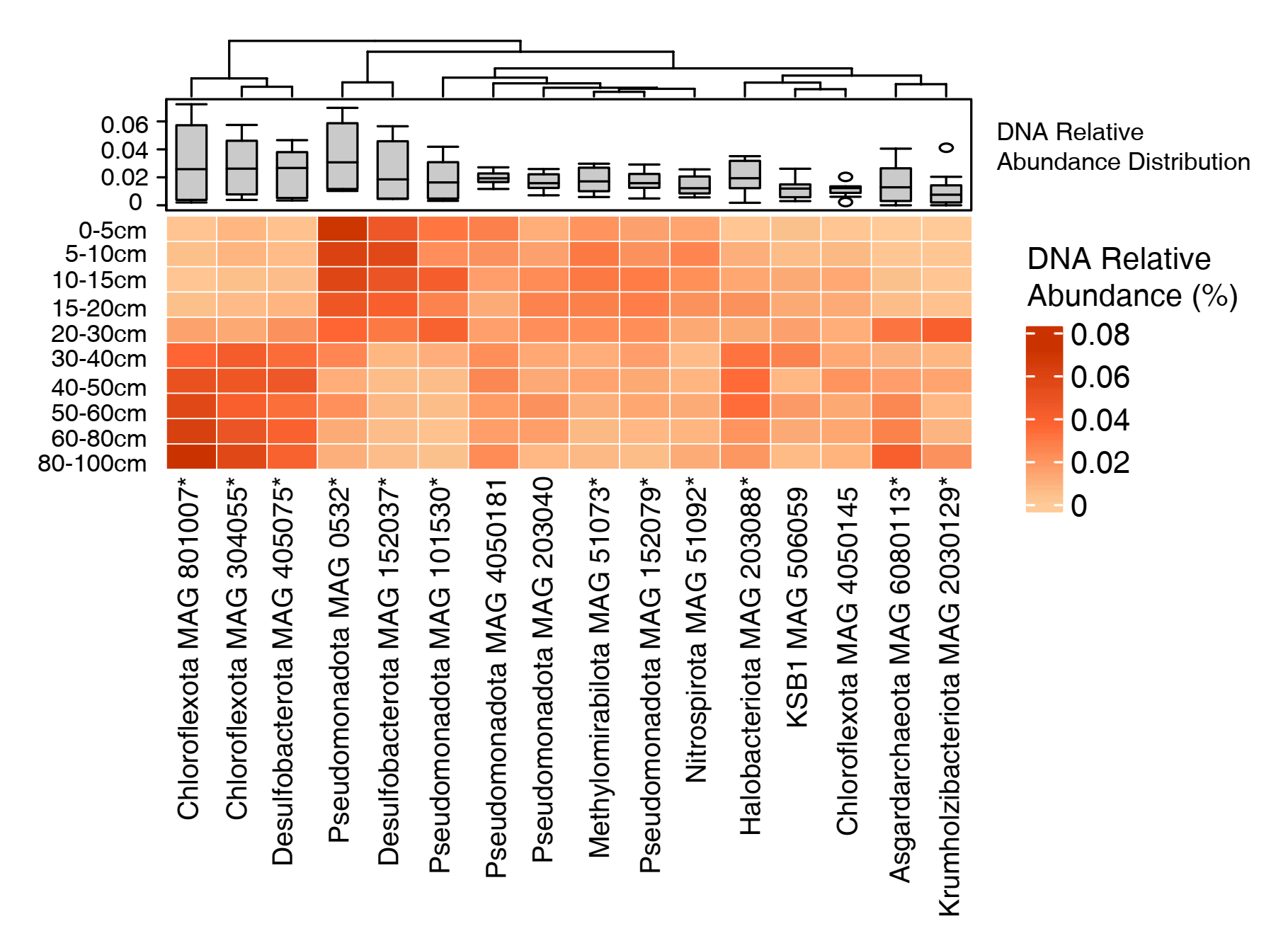


**Fig. S2** Relative abundance of genomes within the 16 microbial populations across all the depths. The heatmap of DNA relative abundance within the 16 microbial populations across the mangrove sediment depths and clustered by *Euclidean Distance.* The boxplot shows their distributions. **p* < 0.05 for the relative abundance between 0-20 cm and 20-100 cm (*t*-test).


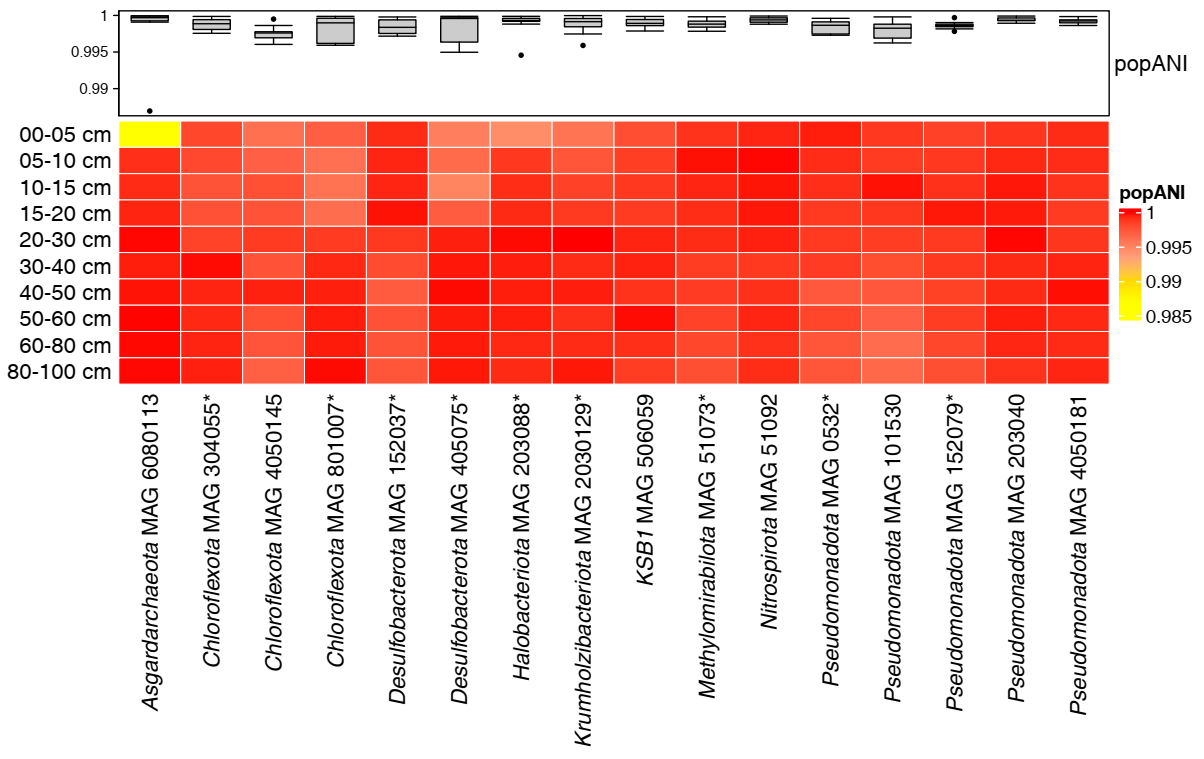


**Fig. S3** Population ANI (popANI) within the 16 microbial populations across all the depths. The heatmap of popANI within the 16 microbial populations across all the depths and clustered by Euclidean Distance*.* The boxplot shows their distributions.


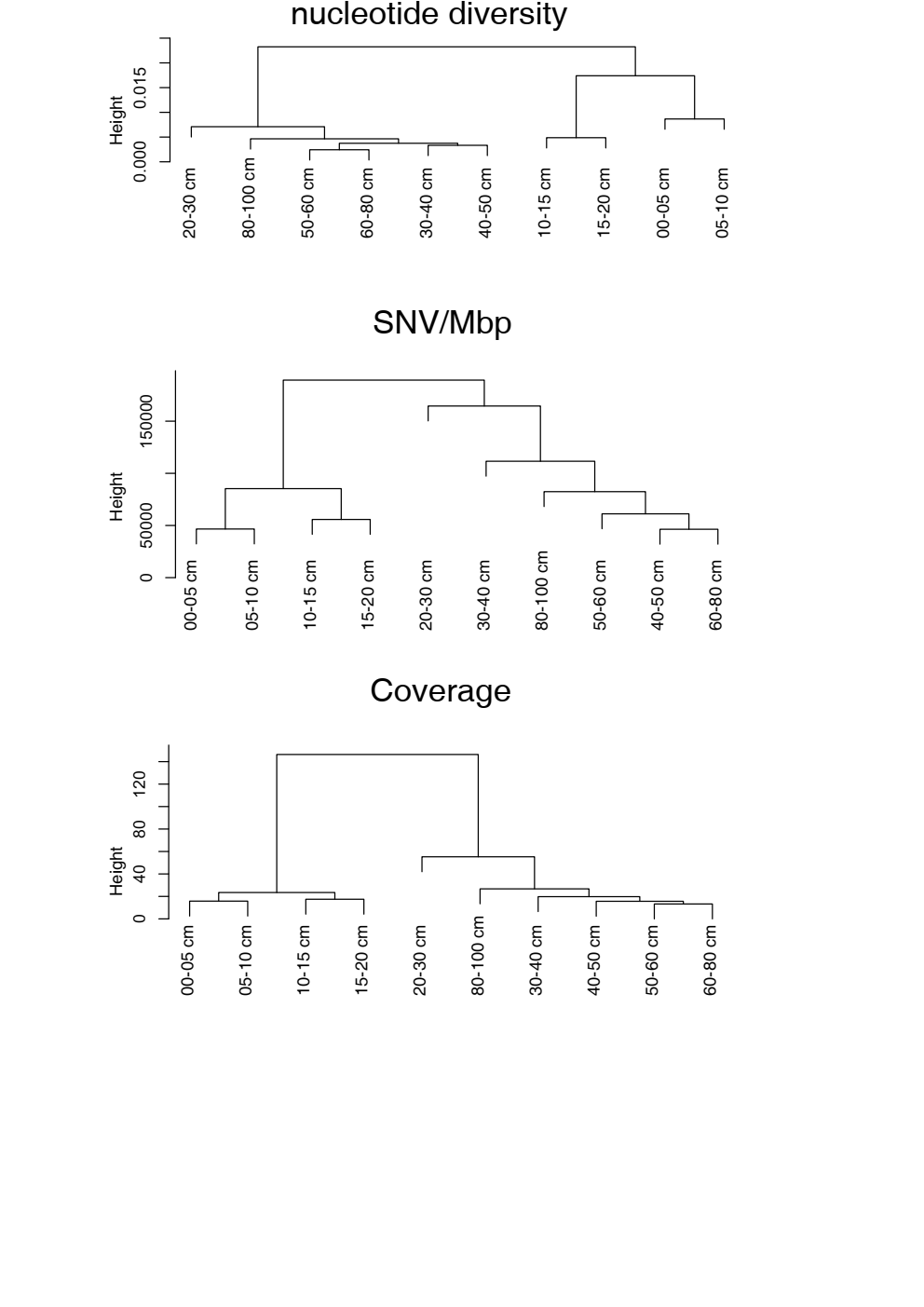


**Fig. S4** The tree plots to the right of these boxplots cluster the features of the microbial community at different depths. The tree was clustered by ward.D2.


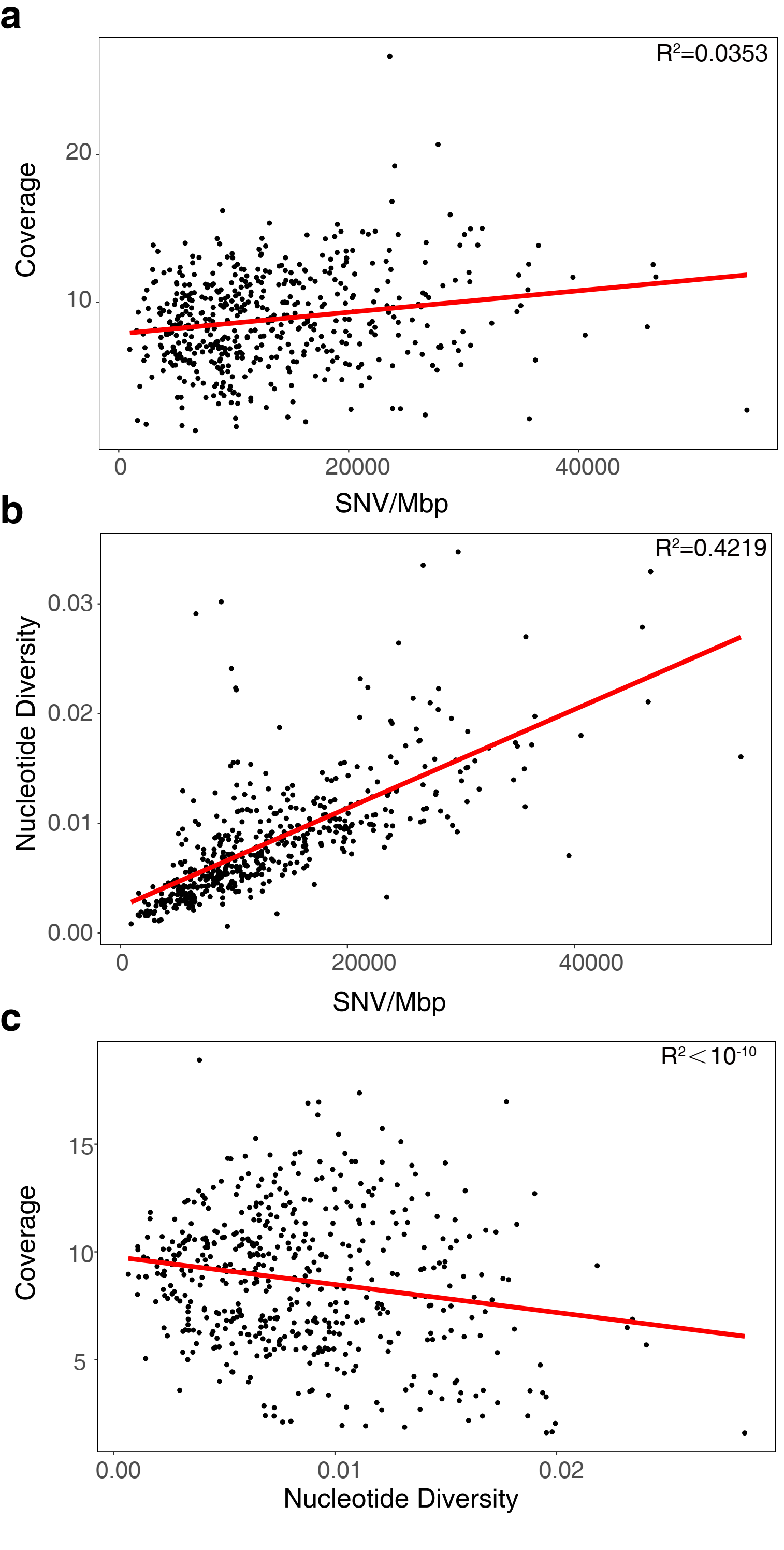


**Fig. S5** The relationships between coverage, nucleotide diversity, and the SNV/Mbp. The relationship between coverage with SNVs/Mbp (linear regression, *R^2^* = 0.0353), nucleotide diversity with the SNVs/Mbp (linear regression, *R^2^* = 0.4219), and nucleotide diversity with coverage (linear regression, *R^2^* < 0.001). Each point represents one gene. SNVs, single nucleotide variants.


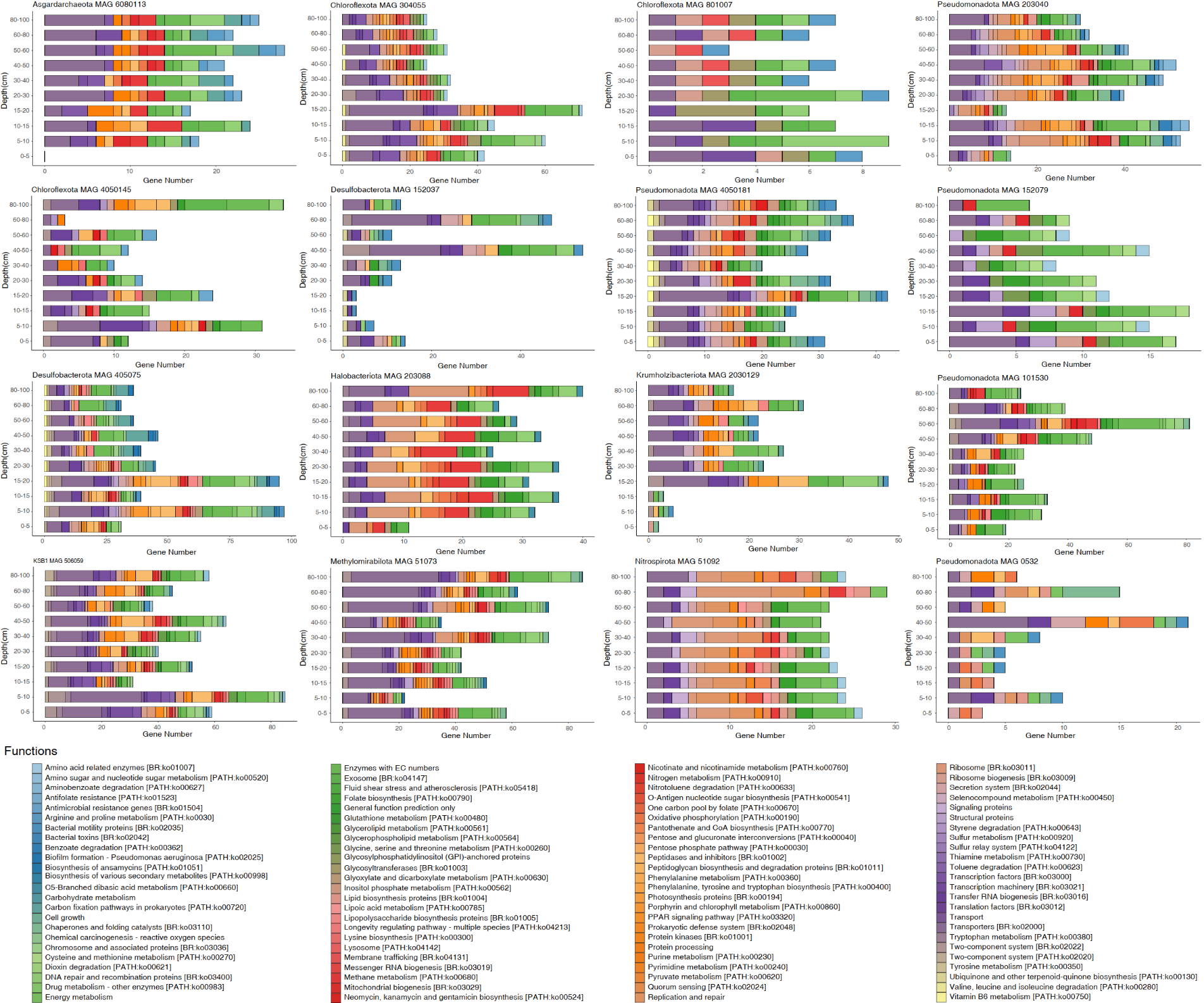


**Fig. S6** Annotation of the high nucleotide diversity genes within the 16 microbial populations. The genes whose nucleotide diversity > 2.5 SDs above the average were selected and annotated against the KEGG database. KEGG, Kyoto Encyclopedia of Genes and Genomes.


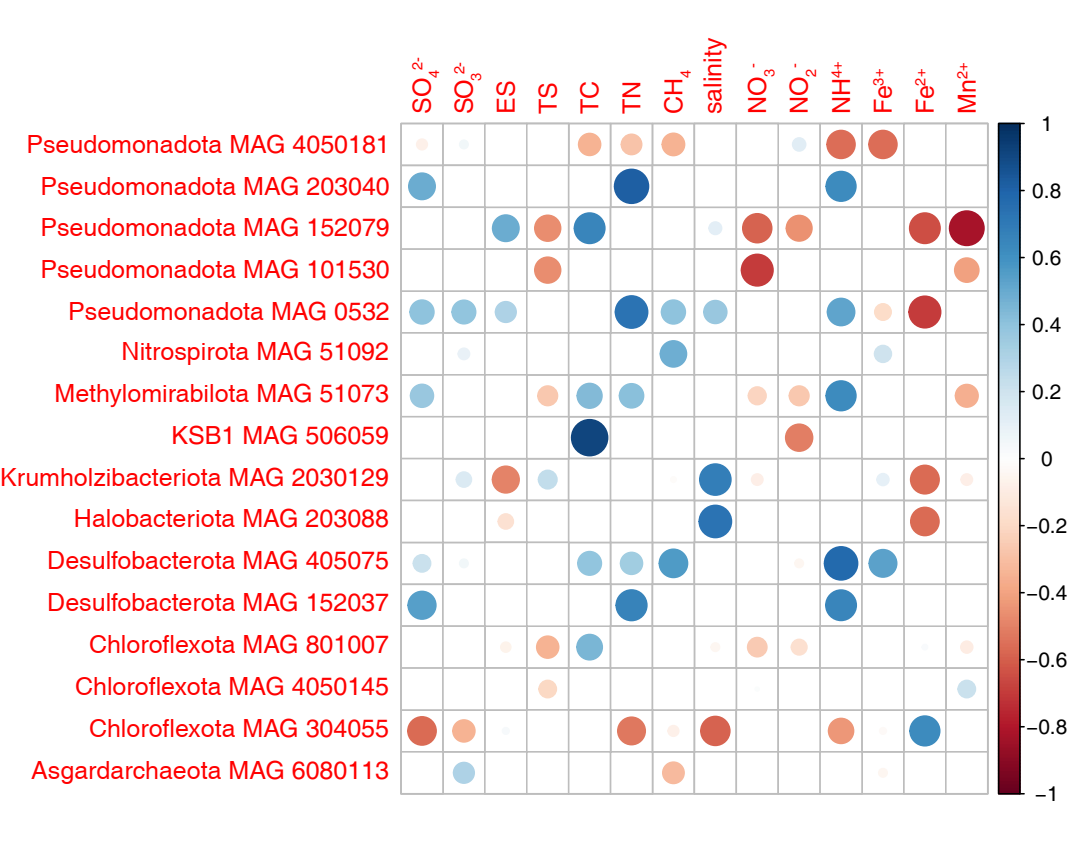


**Fig. S7** The relationships between the 16 microbial populations and environmental factors. The colors represent the positive (red) or negative (blue) relationships. The size of the circle represents the strength of the correlation. The circle is only displayed when *p* < 0.05.


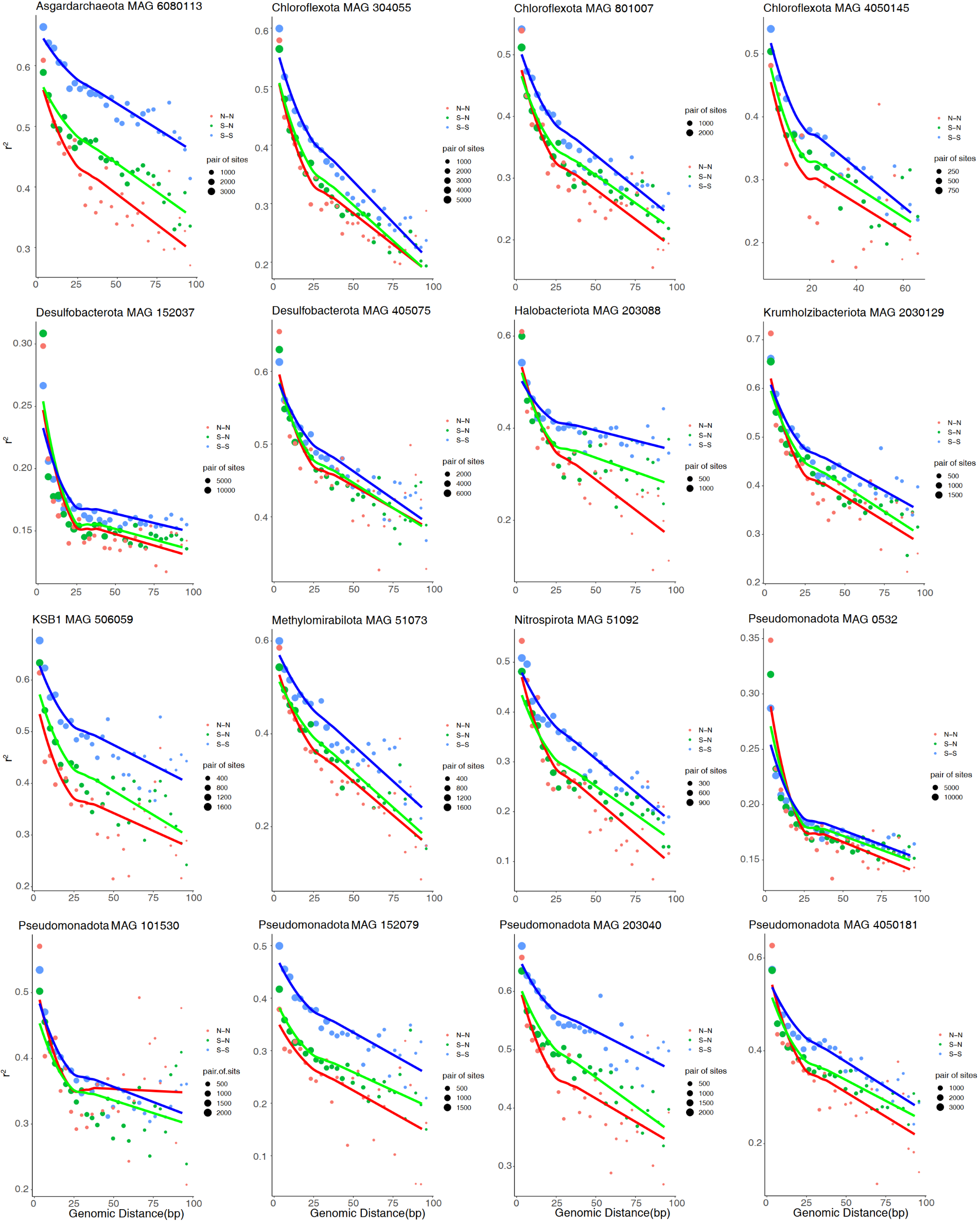


**Fig. S8** Linkage disequilibrium decay over genomic distance within the 16 microbial populations. Each point represents a mean of linkage for the SNVs at that genomic distance. They are divided into nonsynonymous-nonsynonymous linkages, nonsynonymous-synonymous linkages, and synonymous-synonymous linkages. The size of each point represents the number of SNVs that went into calculating the average. SNVs, single nucleotide variants.


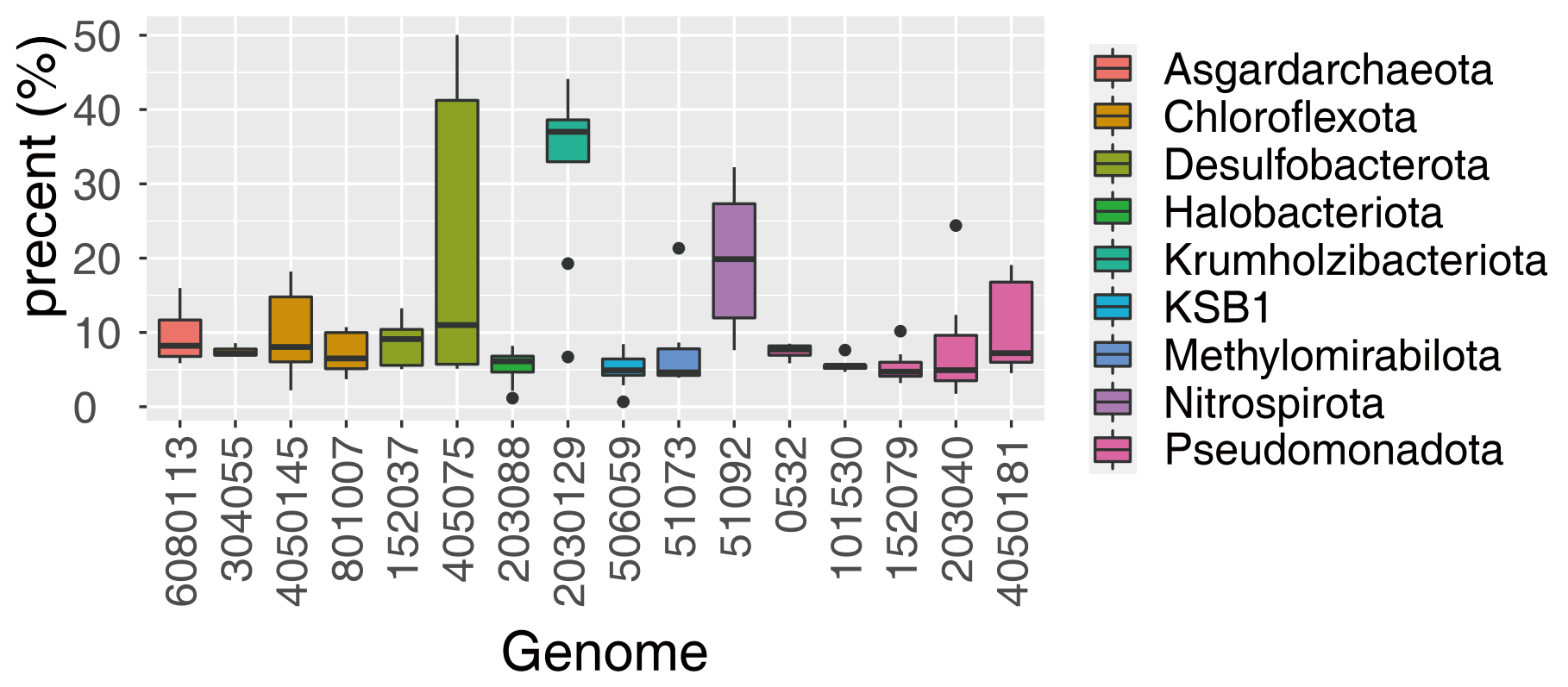


**Fig. S9** The percentage of genes with an average *D'* < 1. Each box represents one microbial population. The colors represent the phylum, and the short line within the box shows the average. Points above the whiskers indicate outliers.


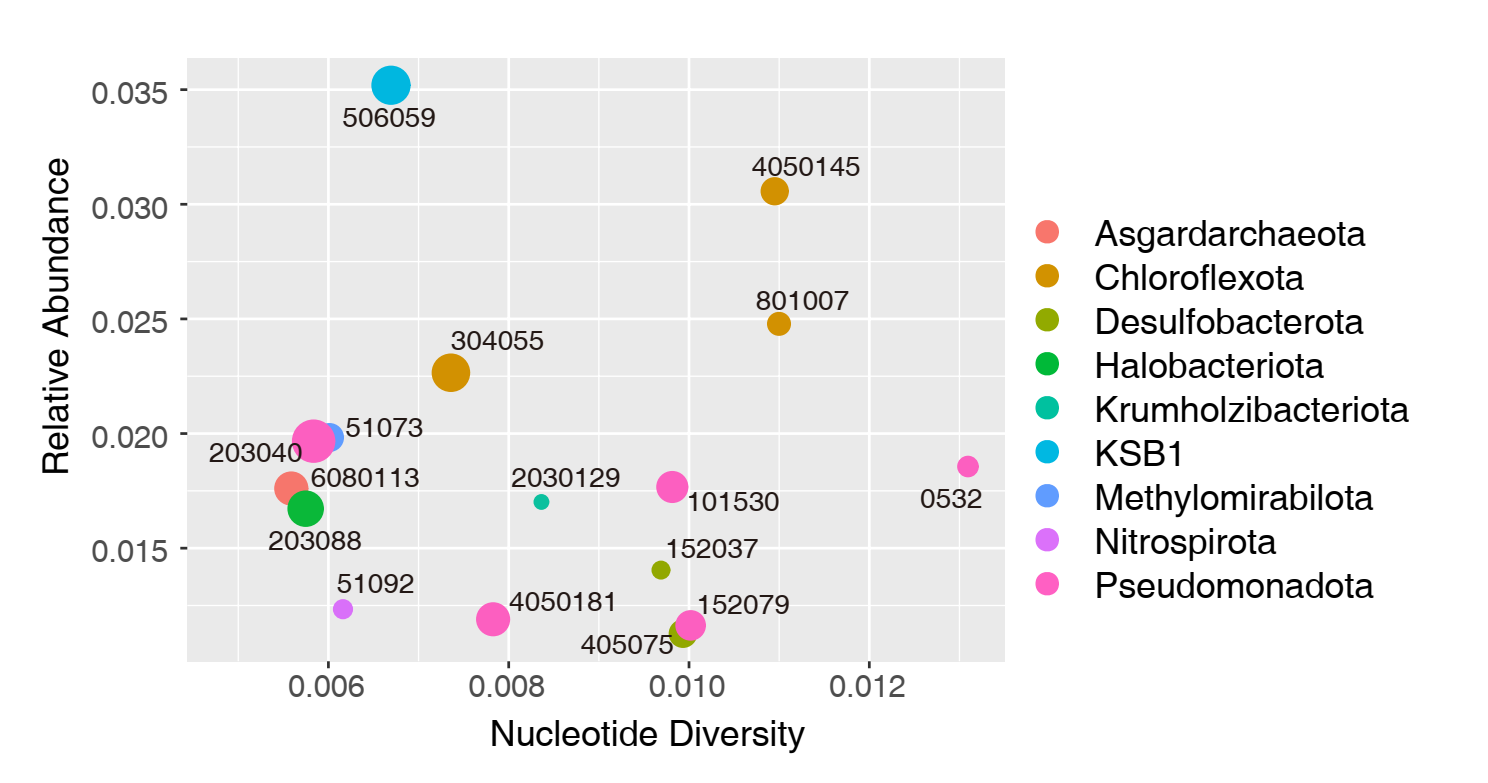


**Fig. S10** Relationships between nucleotide diversity and DNA relative abundance. The relationship between the average nucleotide diversity and relative abundances within the microbial populations across all the depths.

**Table S1** The total base and reads number of each clean read.

| sample_name | depth (cm) | files name (.fastq) | total base count (bp) | total reads number | sample_name | depth (cm) | files name (.fastq) | total base count (bp) | total reads number |
| --- | --- | --- | --- | --- | --- | --- | --- | --- | --- |
| ZH-Q0-5-1 | 0-5 | S0037_1 | 5295564055 | 34606417 | ZH-Q20-30-1 | 20-30 | S0057_1 | 4756616724 | 30862340 |
|  |  | S0037_2 | 5333331009 | 34606417 |  |  | S0057_2 | 4787768730 | 30862340 |
| ZH-Q0-5-2 | 0-5 | S0038_1 | 4869092757 | 31838513 | ZH-Q20-30-2 | 20-30 | S0058_1 | 6242434420 | 40749313 |
|  |  | S0038_2 | 4900312891 | 31838513 |  |  | S0058_2 | 6288348295 | 40749313 |
| ZH-Q0-5-3 | 0-5 | S0039_1 | 5583330651 | 36148697 | ZH-Q20-30-3 | 20-30 | S0059_1 | 9187914086 | 59788364 |
|  |  | S0039_2 | 5603235299 | 36148697 |  |  | S0059_2 | 9265139173 | 59788364 |
| ZH-Q0-5-4 | 0-5 | S0040_1 | 5058451570 | 32883395 | ZH-Q20-30-5 | 20-30 | S0060_1 | 8346705112 | 54654711 |
|  |  | S0040_2 | 5085351212 | 32883395 |  |  | S0060_2 | 8404868962 | 54654711 |
| ZH-Q0-5-5 | 0-5 | S0041_1 | 4675780267 | 30448662 | ZH-Q30-40-2 | 30-40 | S0061_1 | 6799654777 | 44439237 |
|  |  | S0041_2 | 4698705940 | 30448662 |  |  | S0061_2 | 6855286752 | 44439237 |
| ZH-Q5-10-1 | 5-10 | S0042_1 | 5341101289 | 34846093 | ZH-Q30-40-3 | 30-40 | S0062_1 | 6336197055 | 41279353 |
|  |  | S0042_2 | 5373016247 | 34846093 |  |  | S0062_2 | 6370810860 | 41279353 |
| ZH-Q5-10-2 | 5-10 | S0043_1 | 5039984125 | 32881517 | ZH-Q30-40-5 | 30-40 | S0063_1 | 4895219618 | 31941327 |
|  |  | S0043_2 | 5067850762 | 32881517 |  |  | S0063_2 | 4894418496 | 31941327 |
| ZH-Q5-10-3 | 5-10 | S0044_1 | 5196992519 | 33765221 | ZH-Q40-50-2 | 40-50 | S0064_1 | 5403790452 | 35182999 |
|  |  | S0044_2 | 5225064995 | 33765221 |  |  | S0064_2 | 5413645328 | 35182999 |
| ZH-Q5-10-4 | 5-10 | S0045_1 | 6208356601 | 40291210 | ZH-Q40-50-3 | 40-50 | S0065_1 | 5077648750 | 33223736 |
|  |  | S0045_2 | 6246919429 | 40291210 |  |  | S0065_2 | 5099276241 | 33223736 |
| ZH-Q5-10-5 | 5-10 | S0046_1 | 5243728967 | 34126182 | ZH-Q40-50-4 | 40-50 | S0066_1 | 5138011517 | 33685336 |
|  |  | S0046_2 | 5280089900 | 34126182 |  |  | S0066_2 | 5161486297 | 33685336 |
| ZH-Q10-15-1 | 10-15 | S0047_1 | 6471907455 | 42085161 | ZH-Q40-50-5 | 40-50 | S0067_1 | 4751451992 | 30964271 |
|  |  | S0047_2 | 6523094077 | 42085161 |  |  | S0067_2 | 4800463741 | 30964271 |
| ZH-Q10-15-2 | 10-15 | S0048_1 | 8930153324 | 60544329 | ZH-Q50-60-2 | 50-60 | S0068_1 | 6408597437 | 41897125 |
|  |  | S0048_2 | 9022217547 | 60544329 |  |  | S0068_2 | 6430960969 | 41897125 |
| ZH-Q10-15-3 | 10-15 | S0049_1 | 5561999976 | 36238772 | ZH-Q50-60-3 | 50-60 | S0069_1 | 4926083673 | 32088130 |
|  |  | S0049_2 | 5606967231 | 36238772 |  |  | S0069_2 | 4913082693 | 32088130 |
| ZH-Q10-15-4 | 10-15 | S0050_1 | 6300946709 | 41088683 | ZH-Q50-60-4 | 50-60 | S0070_1 | 5269980109 | 34436972 |
|  |  | S0050_2 | 6324612964 | 41088683 |  |  | S0070_2 | 5279054303 | 34436972 |
| ZH-Q10-15-5 | 10-15 | S0051_1 | 5295242326 | 34513120 | ZH-Q50-60-5 | 50-60 | S0071_1 | 5217186716 | 34037053 |
|  |  | S0051_2 | 5329958691 | 34513120 |  |  | S0071_2 | 5219332558 | 34037053 |
| ZH-Q20-30-4 | 20-30 | S0028_1 | 5622667920 | 36818944 | ZH-Q60-80-1 | 60-80 | S0072_1 | 5296862115 | 34589347 |
|  |  | S0028_2 | 5701124457 | 36818944 |  |  | S0072_2 | 5324158890 | 34589347 |
| ZH-Q30-40-1 | 30-40 | S0029_1 | 4929854249 | 32300193 | ZH-Q60-80-2 | 60-80 | S0073_1 | 5136977036 | 33581269 |
|  |  | S0029_2 | 5013181869 | 32300193 |  |  | S0073_2 | 5150244773 | 33581269 |
| ZH-Q30-40-4 | 30-40 | S0030_1 | 5157211918 | 33786874 | ZH-Q60-80-3 | 60-80 | S0074_1 | 4910454393 | 32065315 |
|  |  | S0030_2 | 5227213800 | 33786874 |  |  | S0074_2 | 4937847971 | 32065315 |
| ZH-Q40-50-1 | 40-50 | S0031_1 | 5561702721 | 36453033 | ZH-Q60-80-4 | 60-80 | S0075_1 | 4846000695 | 31668849 |
|  |  | S0031_2 | 5635094830 | 36453033 |  |  | S0075_2 | 4855004887 | 31668849 |
| ZH-Q50-60-1 | 50-60 | S0032_1 | 5299494292 | 34762109 | ZH-Q60-80-5 | 60-80 | S0076_1 | 5181019951 | 33835609 |
|  |  | S0032_2 | 5381766050 | 34762109 |  |  | S0076_2 | 5197358722 | 33835609 |
| ZH-Q15-20-1 | 15-20 | S0052_1 | 5791395064 | 37717955 | ZH-Q80-100-1 | 80-100 | S0077_1 | 5007564190 | 32568181 |
|  |  | S0052_2 | 5830689206 | 37717955 |  |  | S0077_2 | 5008033087 | 32568181 |
| ZH-Q15-20-2 | 15-20 | S0053_1 | 6011269771 | 39142844 | ZH-Q80-100-2 | 80-100 | S0078_1 | 5078118836 | 33208473 |
|  |  | S0053_2 | 6049154892 | 39142844 |  |  | S0078_2 | 5104670136 | 33208473 |
| ZH-Q15-20-3 | 15-20 | S0054_1 | 6688016422 | 43563653 | ZH-Q80-100-3 | 80-100 | S0079_1 | 4876674389 | 31773323 |
|  |  | S0054_2 | 6732757643 | 43563653 |  |  | S0079_2 | 4903937350 | 31773323 |
| ZH-Q15-20-4 | 15-20 | S0055_1 | 5425958751 | 35319288 | ZH-Q80-100-4 | 80-100 | S0080_1 | 5251097613 | 34256599 |
|  |  | S0055_2 | 5480120400 | 35319288 |  |  | S0080_2 | 5269014089 | 34256599 |
| ZH-Q15-20-5 | 15-20 | S0056_1 | 4570412671 | 29899853 | ZH-Q80-100-5 | 80-100 | S0081_1 | 5737822466 | 37304899 |
|  |  | S0056_2 | 4609658023 | 29899853 |  |  | S0081_2 | 5752894980 | 37304899 |

**Table S2** Completeness, contamination, and classification for each reference genome within 16 microbial populations.

| representative genomes | Completeness (%) | Contamination (%) | mean ANI in the population | length | classification |
| --- | --- | --- | --- | --- | --- |
| Asgardarchaeota MAG 6080113 | 94.86 | 2.8 | 0.99844 | 3500707 | d__Archaea;p__Asgardarchaeota;c__Thorarchaeia;o__Thorarchaeales;f__Thorarchaeaceae;g__MP8T-1;s__MP8T-1 sp003345545 |
| Chloroflexota MAG 304055 | 86.36 | 2.73 | 0.99509 | 5059960 | d__Bacteria;p__Chloroflexota;c__Anaerolineae;o__4572-78;f__NAK82;g__NAK82;s__NAK82 sp003130875 |
| Chloroflexota MAG 4050145 | 82.83 | 1.07 | 0.98889 | 3788340 | d__Bacteria;p__Chloroflexota;c__Anaerolineae;o__Thermoflexales;f__Fen-1058;g__Fen-1058;s__Fen-1058 sp003154115 |
| Chloroflexota MAG 801007 | 86.3 | 2.64 | 0.99116 | 1296713 | d__Bacteria;p__Chloroflexota;c__Dehalococcoidia;o__GIF9;f__AB-539-J10;g__;s__ |
| Desulfobacterota MAG 152037 | 90.22 | 1.94 | 0.99462 | 3880070 | d__Bacteria;p__Desulfobacterota;c__Desulfobacteria;o__Desulfobacterales;f__UBA2174;g__UBA2174;s__UBA2174 sp002327445 |
| Desulfobacterota MAG 405075 | 93.76 | 6.77 | 0.99362 | 4256587 | d__Bacteria;p__Desulfobacterota;c__BSN033;o__SM23-61;f__SM23-61;g__SM23-61;s__SM23-61 sp001304105 |
| Halobacteriota MAG 203088 | 98.69 | 3.77 | 0.99528 | 1716877 | d__Archaea;p__Halobacteriota;c__Methanomicrobia;o__Methanomicrobiales;f__Methanoregulaceae;g__UBA9949;s__ |
| Krumholzibacteriota MAG 2030129 | 99.94 | 1.1 | 0.99584 | 4461813 | d__Bacteria;p__Krumholzibacteriota;c__Krumholzibacteria;o__;f__;g__;s__ |
| KSB1 MAG 506059 | 96.64 | 3.66 | 0.98153 | 7766518 | d__Bacteria;p__KSB1;c__UBA2214;o__CR04bin15;f__;g__;s__ |
| Methylomirabilota MAG 51073 | 93.17 | 5.41 | 0.99591 | 5122457 | d__Bacteria;p__Methylomirabilota;c__Methylomirabilia;o__Methylomirabilales;f__;g__;s__ |
| Nitrospirota MAG 51092 | 93.33 | 2.56 | 0.9953 | 2705487 | d__Bacteria;p__Nitrospirota;c__Thermodesulfovibrionia;o__Thermodesulfovibrionales;f__UBA6898;g__UBA6898;s__UBA6898 sp003252075 |
| Pseudomonadota MAG 0532 | 83.09 | 6.49 | 0.99023 | 2518027 | d__Bacteria;p__Pseudomonadota;c__Gammaproteobacteria;o__Thiohalobacterales;f__UBA9214;g__SZUA-186;s__SZUA-186 sp003230545 |
| Pseudomonadota MAG 101530 | 91.78 | 4.95 | 0.99299 | 3507005 | d__Bacteria;p__Pseudomonadota;c__Gammaproteobacteria;o__Burkholderiales;f__;g__;s__ |
| Pseudomonadota MAG 152079 | 87.47 | 2.51 | 0.99401 | 2164652 | d__Bacteria;p__Pseudomonadota;c__Alphaproteobacteria;o__Rhizobiales;f__Methyloligellaceae;g__Methyloceanibacter;s__Methyloceanibacter sp002390155 |
| Pseudomonadota MAG 203040 | 95.47 | 7.26 | 0.99622 | 4968580 | d__Bacteria;p__Pseudomonadota;c__Gammaproteobacteria;o__Steroidobacterales;f__Steroidobacteraceae;g__;s__ |
| Pseudomonadota MAG 4050181 | 97.79 | 4.57 | 0.99518 | 4049059 | d__Bacteria;p__Pseudomonadota;c__Gammaproteobacteria;o__Burkholderiales;f__Burkholderiaceae;g__;s__ |

**Table S3** Summary of key population genetics statistics.

| Microbial Populations | SNV | coverage | nucleotide diversity | D_prime | R2 | Rn/Rs | N:S |
| --- | --- | --- | --- | --- | --- | --- | --- |
| Asgardarchaeota MAG 6080113 | 20531.6 | 10.88512 | 0.005587187 | 0.951354 | 0.375248 | 0.728283 | 0.548191 |
| Chloroflexota MAG 304055 | 47874.2 | 9.319889 | 0.007358816 | 0.978555 | 0.428663 | 0.847225 | 0.485852 |
| Chloroflexota MAG 4050145 | 38029.6 | 5.834262 | 0.010950381 | 0.954418 | 0.302643 | 0.828849 | 0.406397 |
| Chloroflexota MAG 801007 | 15637.4 | 9.509506 | 0.010996839 | 0.964499 | 0.250244 | 0.843129 | 0.485487 |
| Desulfobacterota MAG 152037 | 52078.9 | 9.820979 | 0.009691232 | 0.956865 | 0.188319 | 0.911663 | 0.488449 |
| Desulfobacterota MAG 405075 | 48402.9 | 10.23333 | 0.009932447 | 0.872364 | 0.307231 | 0.962945 | 0.459736 |
| Halobacteriota MAG 203088 | 13797.1 | 10.45202 | 0.005747935 | 0.980789 | 0.403465 | 0.741541 | 0.451751 |
| Krumholzibacteriota MAG 2030129 | 18350.1 | 6.936315 | 0.008363136 | 0.798462 | 0.149709 | 0.879541 | 0.529376 |
| KSB1 MAG 506059 | 43069 | 7.758875 | 0.006693773 | 0.98413 | 0.437019 | 0.730573 | 0.536857 |
| Methylomirabilota MAG 51073 | 37948.9 | 7.545453 | 0.006012872 | 0.97029 | 0.310773 | 0.800917 | 0.493348 |
| Nitrospirota MAG 51092 | 25062 | 9.993143 | 0.006160108 | 0.901307 | 0.20281 | 0.747958 | 0.455667 |
| Pseudomonadota MAG 0532 | 54280 | 10.35202 | 0.013095714 | 0.970243 | 0.221022 | 0.955667 | 0.369363 |
| Pseudomonadota MAG 101530 | 32188 | 7.92269 | 0.009814773 | 0.974597 | 0.34982 | 0.998369 | 0.453404 |
| Pseudomonadota MAG 152079 | 29403.7 | 11.48708 | 0.010021179 | 0.976367 | 0.330595 | 0.672153 | 0.391858 |
| Pseudomonadota MAG 203040 | 38108.2 | 8.175351 | 0.005835325 | 0.970953 | 0.488122 | 0.790776 | 0.462946 |
| Pseudomonadota MAG 4050181 | 51698 | 10.51663 | 0.007827896 | 0.948529 | 0.373711 | 0.83898 | 0.380689 |

**Table S4** The high nucleotide diversity genes.

| index | Pseudomonadota MAG 0532 | Pseudomonadota MAG 101530 | Desulfobacterota MAG 152037 | Pseudomonadota MAG 152079 | Krumholzibacteriota MAG 2030129 | Pseudomonadota MAG 203040 | Halobacteriota MAG 203088 | Chloroflexota MAG 304055 |
| --- | --- | --- | --- | --- | --- | --- | --- | --- |
| K00626 | 0 | 0.01302 | 0.00795 | 0.01101 | 0.00551 | 0.0129 | 0.00995 | 0.00653 |
| K02004 | 0.01292 | 0.01187 | 0.01034 | 0.01089 | 0.00849 | 0.01835 | 0.00762 | 0.0059 |
| K01992 | 0.0176 | 0.01086 | 0.00772 | 0.01045 | 0.00487 | 0.00809 | 0.00381 | 0.00582 |
| K01990 | 0.01236 | 0.01359 | 0.00797 | 0.01398 | 0.00604 | 0.01233 | 0.00312 | 0.00588 |
| K07090 | 0.01433 | 0.00913 | 0.01174 | 0.0123 | 0.00462 | 0.00543 | 0.00323 | 0.01294 |
| K02003 | 0.01233 | 0.00763 | 0.00914 | 0.01606 | 0.00711 | 0.01187 | 0.00413 | 0.00717 |
| K03703 | 0 | 0.00847 | 0.00742 | 0.01267 | 0 | 0.00593 | 0.00272 | 0.0059 |
| K04096 | 0.0137 | 0.0087 | 0.01189 | 0.01061 | 0.00469 | 0.00524 | 0 | 0.00781 |
| K03575 | 0.01451 | 0.01964 | 0.00882 | 0.01143 | 0.0055 | 0.00576 | 0.00535 | 0.01248 |
| K02483 | 0.0114 | 0.00671 | 0.00715 | 0.01105 | 0.0059 | 0.00817 | 0 | 0.01393 |
| K00174 | 0 | 0 | 0.0074 | 0.01156 | 0.00699 | 0.00892 | 0.00226 | 0.00761 |
| K13993 | 0.01317 | 0 | 0.01059 | 0.01473 | 0 | 0.01223 | 0.00359 | 0 |
| K06990 | 0.01538 | 0.01029 | 0.00866 | 0 | 0.0051 | 0 | 0.0039 | 0.01021 |
| K03701 | 0.0156 | 0.01377 | 0.00766 | 0.01547 | 0 | 0 | 0.00285 | 0 |
| K03088 | 0.01347 | 0.01 | 0.0077 | 0 | 0.00599 | 0.01036 | 0 | 0.00936 |
| K02005 | 0.0168 | 0 | 0.00914 | 0.0107 | 0.00624 | 0.00924 | 0 | 0.00575 |
| K01999 | 0.01227 | 0.0078 | 0.00869 | 0.00944 | 0 | 0 | 0.00252 | 0.00753 |
| K01885 | 0.01493 | 0.00835 | 0.00743 | 0.01737 | 0.00443 | 0.00585 | 0.00398 | 0 |
| K00820 | 0.01213 | 0.00806 | 0.0093 | 0.01096 | 0 | 0 | 0.01529 | 0.00669 |
| K00784 | 0.01704 | 0 | 0.00822 | 0.01238 | 0 | 0.00606 | 0.00862 | 0.01534 |
| K12132 | 0 | 0.00875 | 0.00757 | 0 | 0.01018 | 0.00727 | 0.00172 | 0.00578 |
| K04655 | 0 | 0.00781 | 0.00899 | 0 | 0.00547 | 0.00536 | 0.00221 | 0.00826 |
| K04070 | 0 | 0.01368 | 0.00805 | 0 | 0 | 0.00683 | 0.00525 | 0.00944 |
| K00059 | 0 | 0.01163 | 0 | 0.01149 | 0.00425 | 0.02064 | 0 | 0.00805 |
| K07315 | 0.01179 | 0 | 0.01205 | 0 | 0.00622 | 0.00615 | 0 | 0.00579 |
| K07304 | 0.01406 | 0.0077 | 0.00789 | 0.00899 | 0.0052 | 0.00836 | 0.01478 | 0 |
| K07114 | 0.01392 | 0 | 0.0106 | 0 | 0.00576 | 0.00589 | 0 | 0.00881 |
| K06888 | 0.01399 | 0.00839 | 0.00722 | 0 | 0 | 0 | 0.00239 | 0 |
| K06076 | 0.01654 | 0.00721 | 0.00757 | 0 | 0 | 0 | 0 | 0.02643 |
| K04771 | 0.01388 | 0.00863 | 0.01493 | 0.01092 | 0.0053 | 0 | 0 | 0 |
| K04656 | 0.01591 | 0.01296 | 0.00935 | 0 | 0 | 0 | 0.00219 | 0.01006 |
| K04034 | 0.0126 | 0 | 0.00962 | 0 | 0.00565 | 0 | 0.01572 | 0.01629 |
| K03110 | 0.01109 | 0.00663 | 0.00905 | 0 | 0 | 0.01346 | 0.00345 | 0.00634 |
| K02199 | 0.01534 | 0.00833 | 0 | 0.0103 | 0.00402 | 0.01442 | 0 | 0.00791 |
| K01951 | 0.02068 | 0.01486 | 0.00818 | 0.00987 | 0.00533 | 0.00843 | 0.00475 | 0.00629 |
| K01870 | 0.01242 | 0.00699 | 0.01048 | 0.0109 | 0.00478 | 0 | 0.0039 | 0.00625 |
| K01462 | 0.01495 | 0.00716 | 0.00791 | 0.00971 | 0.00387 | 0 | 0.00373 | 0.00632 |
| K00858 | 0.01244 | 0.00706 | 0 | 0.01186 | 0 | 0.0064 | 0.00245 | 0.00592 |
| K00791 | 0.01687 | 0.00713 | 0.00864 | 0.01579 | 0 | 0 | 0 | 0.01052 |
| K00573 | 0.01337 | 0.00898 | 0 | 0.01252 | 0.00354 | 0.00764 | 0.00641 | 0 |
| K10773 | 0 | 0.01079 | 0.00867 | 0.01128 | 0.00538 | 0 | 0.00464 | 0.00966 |
| K04487 | 0 | 0.01 | 0.00859 | 0.00902 | 0.0051 | 0 | 0.00363 | 0.00916 |
| K04069 | 0 | 0.00755 | 0.00811 | 0 | 0.00745 | 0 | 0.00588 | 0.00812 |
| K01874 | 0 | 0.00918 | 0.00729 | 0.00974 | 0.00373 | 0 | 0.00866 | 0 |
| K01845 | 0 | 0.00817 | 0.00965 | 0 | 0.00773 | 0 | 0.00156 | 0.00699 |
| K00611 | 0 | 0.00775 | 0.00837 | 0.01581 | 0.00408 | 0 | 0.00398 | 0 |
| K02034 | 0 | 0 | 0.00946 | 0 | 0.00692 | 0.00528 | 0 | 0.00642 |
| K21929 | 0.01227 | 0.00933 | 0.00736 | 0.01015 | 0 | 0.01016 | 0.00208 | 0.00703 |
| K13038 | 0.01297 | 0.00794 | 0 | 0 | 0.00395 | 0 | 0.00673 | 0.00629 |
| K08309 | 0.01766 | 0.00711 | 0 | 0.01027 | 0.0041 | 0.00654 | 0 | 0.00879 |
| K06915 | 0.01941 | 0.0066 | 0.00924 | 0 | 0 | 0 | 0.00302 | 0.00809 |
| K06147 | 0.01318 | 0.007 | 0 | 0.01023 | 0 | 0 | 0.005 | 0.00825 |
| K05555 | 0.0115 | 0.0086 | 0 | 0.00975 | 0.00938 | 0.01262 | 0 | 0.00858 |
| K04759 | 0.01367 | 0 | 0.00989 | 0 | 0 | 0 | 0.00339 | 0 |
| K03975 | 0.01486 | 0.02127 | 0.00714 | 0 | 0 | 0 | 0.0025 | 0 |
| K03924 | 0.01181 | 0.00767 | 0.01078 | 0 | 0.00515 | 0 | 0 | 0.00591 |
| K03832 | 0.01638 | 0.01473 | 0.00869 | 0 | 0.00495 | 0.01349 | 0 | 0 |
| K03750 | 0.014 | 0 | 0.00732 | 0.01367 | 0 | 0.01037 | 0.00229 | 0 |
| K03630 | 0.01129 | 0.0094 | 0.00928 | 0.01279 | 0 | 0.00992 | 0.00664 | 0 |
| K03601 | 0.01402 | 0.00796 | 0.00805 | 0.01029 | 0.00526 | 0 | 0.00373 | 0.00575 |
| K03470 | 0.01202 | 0.0071 | 0 | 0.01258 | 0.00751 | 0 | 0.01666 | 0 |
| K03424 | 0.01334 | 0.00733 | 0.01155 | 0.01708 | 0.0047 | 0 | 0 | 0 |
| K03320 | 0.013 | 0.01269 | 0 | 0.00998 | 0 | 0.00883 | 0.00174 | 0.01214 |
| K02517 | 0.01908 | 0.00677 | 0.01684 | 0 | 0.00725 | 0 | 0 | 0 |
| K02434 | 0.01097 | 0.00667 | 0.00777 | 0 | 0.00693 | 0 | 0.00325 | 0 |
| K02433 | 0.01167 | 0 | 0 | 0.01447 | 0.004 | 0 | 0.00415 | 0.01394 |
| K02238 | 0.01605 | 0.00873 | 0.00905 | 0 | 0.00535 | 0.00566 | 0 | 0.01175 |
| K02031 | 0.0138 | 0.0096 | 0.0109 | 0 | 0 | 0.00564 | 0 | 0.01263 |
| K01897 | 0.01204 | 0.00731 | 0 | 0 | 0.00371 | 0 | 0 | 0 |
| K01890 | 0.01305 | 0 | 0.00783 | 0.01085 | 0 | 0 | 0.00933 | 0.00956 |
| K01869 | 0.01177 | 0 | 0.00949 | 0.01153 | 0.00547 | 0 | 0.00456 | 0 |
| K01868 | 0.0115 | 0 | 0 | 0 | 0.004 | 0.00644 | 0 | 0.00627 |
| K01810 | 0.01571 | 0.00767 | 0.00991 | 0.01326 | 0.00461 | 0.00772 | 0 | 0 |
| K01207 | 0.01389 | 0.00771 | 0.00801 | 0.01128 | 0.00413 | 0.00697 | 0 | 0 |
| K01011 | 0.01208 | 0.00819 | 0 | 0.0089 | 0.00795 | 0.01196 | 0.00304 | 0 |
| K01006 | 0.01298 | 0 | 0.00842 | 0.00957 | 0 | 0.01083 | 0.00096 | 0 |
| K00797 | 0.01383 | 0 | 0.01234 | 0.01515 | 0.00473 | 0 | 0 | 0 |
| K00688 | 0.01619 | 0.00799 | 0.01006 | 0 | 0 | 0.01211 | 0 | 0 |
| K00600 | 0.01625 | 0.00877 | 0.00932 | 0.00978 | 0 | 0 | 0.00399 | 0.00637 |
| K00057 | 0.01482 | 0.00828 | 0.00813 | 0 | 0.00404 | 0 | 0 | 0 |
| K11690 | 0 | 0.01 | 0.00829 | 0.01053 | 0.00984 | 0.01171 | 0 | 0 |
| K07107 | 0 | 0.00676 | 0.00955 | 0 | 0.00474 | 0 | 0 | 0.0106 |
| K06911 | 0 | 0.01086 | 0.00996 | 0.01913 | 0.00436 | 0.00544 | 0 | 0 |
| K03521 | 0 | 0.00872 | 0.00921 | 0.01146 | 0 | 0 | 0 | 0.00709 |
| K02357 | 0 | 0.0078 | 0.00733 | 0.00982 | 0 | 0.00938 | 0 | 0 |
| K02347 | 0 | 0.01009 | 0.00906 | 0 | 0.00513 | 0 | 0.00216 | 0.00607 |
| K01873 | 0 | 0.00757 | 0.009 | 0.00954 | 0.00354 | 0 | 0.00303 | 0.00719 |
| K01689 | 0 | 0.00795 | 0.00776 | 0.01138 | 0.00471 | 0 | 0.00562 | 0.00737 |
| K00382 | 0 | 0.00852 | 0.01044 | 0.01036 | 0.00747 | 0.00897 | 0 | 0 |
| K08602 | 0 | 0 | 0.01319 | 0.00917 | 0.0047 | 0 | 0 | 0.0092 |
| K03639 | 0 | 0 | 0.00763 | 0.01409 | 0.00708 | 0 | 0.00332 | 0.01095 |
| K02355 | 0 | 0 | 0.00821 | 0.01051 | 0 | 0.00699 | 0 | 0 |
| K00249 | 0 | 0 | 0.01211 | 0 | 0.00645 | 0.00947 | 0 | 0 |
| K00175 | 0 | 0 | 0.00772 | 0 | 0.00827 | 0.01487 | 0.00235 | 0 |
| index | Chloroflexota MAG 4050145 | Pseudomonadota MAG 4050181 | Desulfobacterota MAG 405075 | KSB1 MAG 506059 | Methylomirabilota MAG 51073 | Nitrospirota MAG 51092 | Asgardarchaeota MAG 6080113 | Chloroflexota MAG 801007 |
| K00626 | 0.00868 | 0.01327 | 0.01109 | 0.00755 | 0.00845 | 0.00659 | 0.00428 | 0.00838 |
| K02004 | 0 | 0.00917 | 0.01547 | 0.02136 | 0.0062 | 0.00969 | 0.0053 | 0 |
| K01992 | 0 | 0.00741 | 0.00627 | 0.0084 | 0.01615 | 0 | 0.01208 | 0.01126 |
| K01990 | 0 | 0.01252 | 0.00772 | 0.0085 | 0.01551 | 0.00591 | 0.00427 | 0 |
| K07090 | 0 | 0.01307 | 0 | 0 | 0.00946 | 0.016 | 0.00476 | 0.00728 |
| K02003 | 0 | 0.00827 | 0 | 0.01139 | 0.00788 | 0.00775 | 0.00504 | 0 |
| K03703 | 0 | 0.00751 | 0.00823 | 0.00523 | 0.00599 | 0.00632 | 0.00727 | 0.01107 |
| K04096 | 0 | 0.00812 | 0.00719 | 0.00837 | 0 | 0.0068 | 0 | 0.00694 |
| K03575 | 0 | 0.01185 | 0.00768 | 0.00795 | 0 | 0 | 0.0107 | 0 |
| K02483 | 0.01186 | 0.01915 | 0 | 0.01125 | 0 | 0.00687 | 0 | 0.00811 |
| K00174 | 0 | 0 | 0.01477 | 0.00536 | 0.00979 | 0.00722 | 0.00539 | 0.01023 |
| K13993 | 0 | 0.01062 | 0.01163 | 0.00568 | 0.01207 | 0.00622 | 0 | 0.01212 |
| K06990 | 0 | 0.0075 | 0.01054 | 0.00661 | 0.01038 | 0.00658 | 0 | 0 |
| K03701 | 0 | 0.00734 | 0.01564 | 0.0106 | 0 | 0.0066 | 0.00406 | 0.01738 |
| K03088 | 0 | 0.01396 | 0 | 0.01454 | 0.01471 | 0.00745 | 0 | 0.01661 |
| K02005 | 0.02095 | 0 | 0.01113 | 0.01156 | 0.01401 | 0.00637 | 0 | 0 |
| K01999 | 0 | 0.00925 | 0.015 | 0.00663 | 0.01289 | 0 | 0 | 0.01059 |
| K01885 | 0 | 0.00898 | 0.01071 | 0 | 0.00553 | 0.00621 | 0 | 0 |
| K00820 | 0 | 0 | 0.00826 | 0.01077 | 0 | 0.00991 | 0.00423 | 0.01102 |
| K00784 | 0 | 0.009 | 0.00714 | 0.01019 | 0.00521 | 0 | 0.00746 | 0 |
| K12132 | 0.02353 | 0.01288 | 0.01005 | 0.01702 | 0.01515 | 0 | 0 | 0 |
| K04655 | 0 | 0.00935 | 0.00638 | 0.00697 | 0.00589 | 0 | 0.00529 | 0 |
| K04070 | 0 | 0.0138 | 0.0063 | 0.00901 | 0 | 0.00716 | 0.00544 | 0.01012 |
| K00059 | 0 | 0.01265 | 0.0114 | 0.00934 | 0.01634 | 0.00614 | 0 | 0.00871 |
| K07315 | 0.01245 | 0.00935 | 0 | 0.00965 | 0 | 0.0059 | 0 | 0.01036 |
| K07304 | 0 | 0.01 | 0 | 0 | 0 | 0.00797 | 0.00458 | 0 |
| K07114 | 0.01658 | 0.00944 | 0.00944 | 0.01616 | 0.00737 | 0 | 0 | 0 |
| K06888 | 0 | 0.01376 | 0.011 | 0.00836 | 0 | 0.01086 | 0.00705 | 0.00954 |
| K06076 | 0 | 0.01192 | 0.00742 | 0.01059 | 0.01244 | 0.00802 | 0 | 0.01031 |
| K04771 | 0.01165 | 0 | 0.00809 | 0.01436 | 0.00852 | 0.01108 | 0 | 0 |
| K04656 | 0 | 0.00879 | 0.00767 | 0 | 0.00883 | 0 | 0.00863 | 0.01022 |
| K04034 | 0 | 0 | 0.01405 | 0.00746 | 0.01029 | 0.00834 | 0.01082 | 0 |
| K03110 | 0 | 0 | 0.00621 | 0 | 0 | 0.00768 | 0.0089 | 0.00707 |
| K02199 | 0 | 0.00863 | 0.00839 | 0 | 0.01061 | 0.00832 | 0 | 0 |
| K01951 | 0 | 0.01391 | 0.00941 | 0 | 0 | 0 | 0 | 0 |
| K01870 | 0 | 0.00718 | 0 | 0 | 0 | 0 | 0.00477 | 0.006 |
| K01462 | 0 | 0 | 0.00805 | 0 | 0 | 0 | 0.00508 | 0.01955 |
| K00858 | 0.01601 | 0 | 0.00616 | 0.00528 | 0 | 0 | 0.00739 | 0 |
| K00791 | 0 | 0.00872 | 0.00915 | 0.00532 | 0.00509 | 0.00747 | 0 | 0 |
| K00573 | 0 | 0.01522 | 0.01247 | 0 | 0.00714 | 0.009 | 0 | 0 |
| K10773 | 0 | 0.0072 | 0.00888 | 0.00896 | 0.00535 | 0 | 0 | 0 |
| K04487 | 0 | 0 | 0.01428 | 0.01077 | 0.00773 | 0 | 0 | 0.01725 |
| K04069 | 0 | 0.0073 | 0.01166 | 0 | 0 | 0.00623 | 0.005 | 0.00854 |
| K01874 | 0 | 0.00957 | 0.01136 | 0 | 0 | 0.0072 | 0.00594 | 0.00772 |
| K01845 | 0 | 0.00837 | 0 | 0.00807 | 0.01109 | 0.00585 | 0.00437 | 0 |
| K00611 | 0 | 0.00798 | 0.01882 | 0.00753 | 0 | 0.00678 | 0.00762 | 0 |
| K02034 | 0.00989 | 0.00763 | 0.01294 | 0.00634 | 0.00877 | 0.00647 | 0 | 0 |
| K21929 | 0 | 0.01454 | 0 | 0 | 0.00608 | 0 | 0 | 0 |
| K13038 | 0 | 0.00885 | 0.01034 | 0 | 0 | 0.00615 | 0.0057 | 0 |
| K08309 | 0 | 0.0085 | 0 | 0 | 0.00605 | 0.00579 | 0 | 0 |
| K06915 | 0 | 0.01335 | 0.00952 | 0.0052 | 0 | 0 | 0.00416 | 0 |
| K06147 | 0 | 0.0103 | 0 | 0.00616 | 0.00554 | 0 | 0.00475 | 0 |
| K05555 | 0 | 0.01688 | 0.00976 | 0 | 0 | 0 | 0.01165 | 0 |
| K04759 | 0 | 0.01286 | 0.00872 | 0.00896 | 0.00543 | 0.01159 | 0.00441 | 0 |
| K03975 | 0 | 0.00724 | 0.01172 | 0.0102 | 0.00789 | 0.00672 | 0 | 0 |
| K03924 | 0 | 0.0111 | 0.00829 | 0.00569 | 0 | 0 | 0.00561 | 0 |
| K03832 | 0 | 0.01342 | 0 | 0.00749 | 0.00495 | 0.01067 | 0 | 0 |
| K03750 | 0 | 0.00716 | 0.00736 | 0 | 0 | 0 | 0.00572 | 0.00804 |
| K03630 | 0 | 0.00804 | 0 | 0.00685 | 0 | 0 | 0 | 0.00845 |
| K03601 | 0 | 0.01315 | 0.01031 | 0 | 0 | 0 | 0 | 0 |
| K03470 | 0 | 0.00979 | 0.01079 | 0.00739 | 0 | 0 | 0.00532 | 0 |
| K03424 | 0 | 0 | 0.00599 | 0 | 0.01216 | 0 | 0.00506 | 0.00796 |
| K03320 | 0 | 0 | 0.00983 | 0 | 0.00595 | 0.00675 | 0 | 0 |
| K02517 | 0 | 0.00738 | 0.00829 | 0.01073 | 0.00571 | 0.00631 | 0 | 0 |
| K02434 | 0 | 0 | 0.0077 | 0.00799 | 0 | 0.0089 | 0 | 0.00743 |
| K02433 | 0 | 0 | 0.01123 | 0 | 0.00527 | 0.00683 | 0 | 0.00635 |
| K02238 | 0 | 0 | 0.00705 | 0 | 0.0056 | 0.0086 | 0 | 0 |
| K02031 | 0.01604 | 0.0111 | 0.01274 | 0 | 0 | 0 | 0.00748 | 0 |
| K01897 | 0 | 0.00888 | 0.01123 | 0.00881 | 0 | 0.00684 | 0.00406 | 0.0194 |
| K01890 | 0 | 0.01093 | 0.00702 | 0 | 0.00603 | 0.00624 | 0 | 0 |
| K01869 | 0 | 0.00714 | 0.01096 | 0.00707 | 0.00613 | 0 | 0 | 0 |
| K01868 | 0 | 0 | 0.0086 | 0.00727 | 0.00591 | 0.00665 | 0.00513 | 0 |
| K01810 | 0 | 0.00799 | 0.01158 | 0 | 0 | 0.00769 | 0 | 0 |
| K01207 | 0 | 0.01036 | 0 | 0 | 0.00526 | 0.00735 | 0 | 0 |
| K01011 | 0 | 0.00944 | 0 | 0.01199 | 0.00819 | 0 | 0 | 0 |
| K01006 | 0 | 0 | 0.00916 | 0.00521 | 0.01459 | 0 | 0.00888 | 0 |
| K00797 | 0 | 0.00816 | 0.00759 | 0.01765 | 0.00713 | 0.00702 | 0 | 0 |
| K00688 | 0 | 0.00729 | 0.01027 | 0.00657 | 0.00647 | 0.0131 | 0 | 0 |
| K00600 | 0 | 0.0077 | 0 | 0 | 0.00582 | 0 | 0.00983 | 0 |
| K00057 | 0 | 0.00742 | 0.00613 | 0.00525 | 0.00779 | 0 | 0 | 0.00764 |
| K11690 | 0 | 0.01009 | 0.0153 | 0 | 0.01182 | 0 | 0 | 0.01277 |
| K07107 | 0 | 0.00766 | 0.01314 | 0.00728 | 0.0079 | 0.00905 | 0 | 0 |
| K06911 | 0 | 0.00895 | 0.00816 | 0.0067 | 0.00721 | 0 | 0 | 0 |
| K03521 | 0 | 0 | 0.01224 | 0.00576 | 0.00824 | 0 | 0.0049 | 0.00627 |
| K02357 | 0 | 0 | 0.00607 | 0.00636 | 0.00499 | 0.00683 | 0 | 0.00746 |
| K02347 | 0 | 0.01071 | 0.01203 | 0.00971 | 0 | 0 | 0.00931 | 0 |
| K01873 | 0 | 0.0173 | 0.00955 | 0.00998 | 0 | 0 | 0 | 0 |
| K01689 | 0 | 0.01919 | 0.00909 | 0 | 0.0058 | 0 | 0 | 0 |
| K00382 | 0 | 0 | 0.01014 | 0.00784 | 0.00559 | 0.00607 | 0 | 0 |
| K08602 | 0 | 0 | 0.01002 | 0.00905 | 0.00756 | 0.00651 | 0.00438 | 0 |
| K03639 | 0 | 0.00764 | 0 | 0 | 0.00641 | 0.00579 | 0.00525 | 0 |
| K02355 | 0 | 0.00721 | 0.01148 | 0.00629 | 0.01719 | 0.00622 | 0 | 0.00748 |
| K00249 | 0 | 0.01006 | 0.0164 | 0.00736 | 0.01496 | 0.00699 | 0.00414 | 0 |
| K00175 | 0 | 0 | 0.01976 | 0.00897 | 0.01023 | 0 | 0.00816 | 0.00965 |

**Table S5** The environmental factors in 0-100 cm mangrove sediments.

|  | 0-5 cm | 5-10 cm | 10-15 cm | 15-20 cm | 20-30 cm | 30-40 cm | 40-50 cm | 50-60 cm | 60-80 cm | 80-100 cm |
| --- | --- | --- | --- | --- | --- | --- | --- | --- | --- | --- |
| SO_4_^2-^ (mg/L) | 596.2 | 260.8 | 155.3 | 88.2 | 115.0 | 95.5 | 133.5 | 55.5 | 64.0 | 30.5 |
| AVS (mmol/g) | 13.9 | 22.4 | 47.1 | 62.8 | 58.7 | 36.4 | 29.2 | 13.5 | 8.4 | 9.8 |
| TS (mmol/g) | 29.4 | 25.7 | 22.8 | 24.0 | 26.2 | 28.4 | 31.8 | 33. | 34.7 | 32.1 |
| TC (%) | 3.2 | 3.6 | 3.6 | 3.4 | 3.1 | 2.7 | 2.4 | 2.4 | 2.4 | 1.8 |
| TN (%) | 0.3 | 0.3 | 0.3 | 0.3 | 0.3 | 0.2 | 0.2 | 0.2 | 0.2 | 0.1 |
| CH_4_ (ppm) | 8.5 | 38.2 | 10.9 | 16.3 | 5.9 | 8.1 | 15.9 | 6.3 | 11.6 | 7.6 |
| Salinity (%) | 1.3 | 0.9 | 1.0 | 0.9 | 0.9 | 0.9 | 0.9 | 0.8 | 0.9 | 0.9 |
| NO_3_^-^ (μg N/kg) | 133.9 | 139.6 | 88.3 | 101.1 | 229.9 | 148.4 | 213.1 | 227.9 | 267.9 | 294.9 |
| NO_2_^-^ (μg N/kg) | 91.8 | 58.9 | 86.2 | 47.1 | 81.0 | 85.1 | 95.5 | 89.7 | 86.9 | 103.9 |
| NH_4_^+^ (mg/g) | 0.7 | 0.9 | 0.6 | 0.4 | 0.4 | 0.4 | 0.4 | 0.5 | 0.5 | 0.4 |
| Fe^3+^ (mg/g) | 10.8 | 10.9 | 12.1 | 8.8 | 8.0 | 8.4 | 9.1 | 10.5 | 10.3 | 15.1 |
| Fe^2+^ (mg/g) | 245.2 | 580.5 | 707.4 | 537.9 | 573.4 | 753.9 | 647.7 | 739.7 | 1058.5 | 1091.6 |
| Mn^2+^ (mg/g) | 0.05 | 0.05 | 0.03 | 0.04 | 0.03 | 0.05 | 0.05 | 0.03 | 0.11 | 0.09 |
| ES (μmol/g) | 4394.1 | 5131.5 | 4045.5 | 3265.9 | 3147.3 | 3165.1 | 2940.1 | 2751.8 | 1080.9 | 2432.3 |

**Table S6** Annotations and key genetic statistics of highly differentiated genes.

| microbial populations | gene | ko_number | fst | snv number | nucleotide diversity | dn/ds | pn/ps |
| --- | --- | --- | --- | --- | --- | --- | --- |
| Asgardarchaeota MAG 6080113 | NODE_2_length_196012_cov_6.433414_131 |  | 0.538462 | 11 | 0.0019136 | 0 | 0.0099726 |
|  | NODE_4_length_171049_cov_7.433100_55 |  | 0.538462 | 36 | 0.0049908 | 0 | 0.0581896 |
|  | NODE_7_length_131267_cov_6.955195_6 | K01474 | 0.538462 | 43 | 0.0047655 | 0.0312759 | 0.0860352 |
|  | NODE_10_length_120127_cov_7.312686_89 |  | 0.538462 | 7 | 0.0009851 | 0 | 0.0116488 |
|  | NODE_11_length_120066_cov_6.213236_46 |  | 0.53012 | 7 | 0.0060808 | 0 | 0.3573826 |
|  | NODE_15_length_98588_cov_6.374547_11 |  | 0.538462 | 2 | 0.0030942 | 0 | 0.0466867 |
|  | NODE_15_length_98588_cov_6.374547_15 |  | 0.519231 | 48 | 0.00593 | 0 | 0.088215 |
|  | NODE_15_length_98588_cov_6.374547_42 |  | 1 | 7 | 0.0016966 | 0.1244086 | 0.0848598 |
|  | NODE_22_length_57954_cov_6.496536_43 |  | 1 | 7 | 0.0065819 | 0 | 0.3297258 |
|  | NODE_46_length_17675_cov_5.449483_8 | K00767 | 0.519231 | 29 | 0.0047375 | 0 | 0.0555129 |
| Chloroflexota MAG 304055 | k141_824955_length_28951_cov_15.0209_24 | K00335 | 0.473487 | 49 | 0.0061033 | 0.0933226 | 0.0329216 |
|  | k141_309431_length_9809_cov_15.1710_5 | K01271 | 0.491018 | 32 | 0.0080151 | 0 | 0.1200952 |
|  | k141_2205504_length_8358_cov_14.5420_7 | K00925 | 0.453642 | 24 | 0.0056844 | 0 | 0.0290349 |
|  | k141_4223550_length_7962_cov_12.1454_5 | K11995 | 0.491018 | 24 | 0.0093853 | 0.0319948 | 0.0491017 |
|  | k141_4721550_length_7868_cov_14.5706_8 |  | 0.473684 | 7 | 0.0035669 | 0 | 0.0835066 |
|  | k141_2716860_length_7583_cov_16.5930_1 |  | 0.453642 | 35 | 0.0048488 | 0 | 0.1521081 |
|  | k141_7060041_length_5729_cov_8.2287_6 | K00230 | 1 | 23 | 0.0076052 | 0.065798 | 0.2571607 |
|  | k141_234508_length_5685_cov_14.8983_4 | K03177 | 0.454545 | 22 | 0.0064732 | 0.035 | 0.1553584 |
|  | k141_4724641_length_5588_cov_13.8880_3 |  | 0.473684 | 15 | 0.0020225 | 0 | 0.0217635 |
|  | k141_3520340_length_4523_cov_17.6052_4 |  | 0.454545 | 7 | 0.0043814 | 0 | 0.2076271 |
|  | k141_3553560_length_3569_cov_9.5067_2 | K01687 | 0.4713807 | 43 | 0.01423 | 0.0729367 | 0.1704348 |
|  | k141_5554335_length_3412_cov_16.0000_2 | K12132 | 0.473684 | 47 | 0.0110086 | 0 | 0.0616749 |
|  | k141_4667101_length_2884_cov_10.0004_3 | K03797 | 0.531593 | 25 | 0.0099544 | 0 | 0.0499819 |
|  | k141_4715391_length_2246_cov_11.9905_2 |  | 0.538462 | 34 | 0.005948 | 0 | 0.0232893 |
|  | k141_3177154_length_1726_cov_7.6953_1 |  | 0.836478 | 31 | 0.0075928 | 0.25992 | 0.0760507 |
| Chloroflexota MAG 801007 | NODE_2_length_34927_cov_7.740689_1 | K00554 | 0.690667 | 13 | 0.0028663 | 0 | 0.1242857 |
|  | NODE_2_length_34927_cov_7.740689_14 | K14534 | 0.565957 | 67 | 0.0044071 | 0 | 0.0597826 |
|  | NODE_3_length_33527_cov_6.142627_5 |  | 0.565957 | 11 | 0.0069398 | 0 | 0.0219543 |
|  | NODE_36_length_9763_cov_7.180467_8 |  | 0.5758185 | 48 | 0.0071383 | 0 | 0.2270964 |
|  | NODE_42_length_9259_cov_5.134502_8 | K01056 | 0.836478 | 19 | 0.0041348 | 0 | 0.3554685 |
|  | NODE_52_length_6983_cov_7.394150_1 | K05982 | 0.565957 | 13 | 0.0077604 | 0 | 0.2911588 |
|  | NODE_64_length_5786_cov_4.950079_4 |  | 0.628312 | 42 | 0.0065797 | 0 | 0.0698067 |
|  | NODE_89_length_4663_cov_4.984518_3 | K10907 | 0.5708883 | 37 | 0.0081953 | 0.0059081 | 0.0888979 |
|  | NODE_100_length_4084_cov_4.301722_2 |  | 0.565957 | 1 | 0.0101373 | 0.0722581 | 0.1623104 |
|  | NODE_119_length_3123_cov_6.820420_1 | K00830 | 0.6024495 | 50 | 0.0092014 | 0.046443 | 0.1999838 |
| Chloroflexota MAG 4050145 | NODE_4_length_27140_cov_2.626196_3 | K02906 | 0.384615 | 1 | 0.0044236 | 0 | 0.0042888 |
|  | NODE_5_length_25642_cov_2.175136_18 | K04096 | 0.384615 | 13 | 0.013965 | 0 | 0.0374344 |
|  | NODE_33_length_16086_cov_2.010869_13 |  | 0.49884 | 2 | 0.0069974 | 0.0112842 | 0.0894038 |
|  | NODE_45_length_14697_cov_1.883721_3 |  | 0.453853 | 23 | 0.0136133 | 0.0174765 | 0.0299879 |
|  | NODE_61_length_13117_cov_1.139328_6 |  | 0.428571 | 1 | 0.008308 | 0 | 0.0205079 |
|  | NODE_108_length_9455_cov_1.460168_5 |  | 0.369318 | 6 | 0.0070601 | 0.2634093 | 0.1506665 |
|  | NODE_112_length_9430_cov_2.249118_1 |  | 0.4141415 | 2 | 0.0138695 | 0.0225778 | 0.1237416 |
|  | NODE_126_length_8920_cov_1.799729_2 | K07403 | 0.453642 | 8 | 0.0116545 | 0 | 0.1176403 |
|  | NODE_127_length_8845_cov_2.283645_4 | K01733 | 0.491018 | 37 | 0.0111076 | 0.0326684 | 0.0358284 |
|  | NODE_146_length_7913_cov_1.770801_7 | K22452 | 0.4 | 28 | 0.0126523 | 0.1817314 | 0.4614726 |
|  | NODE_168_length_7102_cov_1.794875_3 | K15975 | 0.391026 | 13 | 0.0133874 | 0.065192 | 0.2688199 |
|  | NODE_170_length_7025_cov_1.171535_1 | K07484 | 0.384615 | 1 | 0.0159171 | 0.0509247 | 0.1415426 |
|  | NODE_217_length_5967_cov_1.331070_3 |  | 0.384615 | 8 | 0.0111454 | 0 | 0.401531 |
|  | NODE_229_length_5704_cov_1.467129_4 | K06402 | 0.384615 | 1 | 0.0061951 | 0 | 0.1786429 |
|  | NODE_241_length_5543_cov_1.892975_6 |  | 0.384615 | 1 | 0.0101364 | 0.3011643 | 0.3281247 |
|  | NODE_247_length_5348_cov_1.986153_3 | K02884 | 0.384615 | 1 | 0.0021499 | 0 | 0.0417186 |
|  | NODE_334_length_3971_cov_1.524397_5 | K00249 | 0.459542 | 3 | 0.017519 | 0.0053402 | 0.0516369 |
|  | NODE_363_length_3642_cov_3.409537_4 | K03631 | 1 | 20 | 0.0066506 | 0.0200917 | 0.088852 |
|  | NODE_411_length_3031_cov_1.968517_2 | K07407 | 0.384615 | 3 | 0.0125084 | 0.1330834 | 0.2915798 |
|  | NODE_421_length_2916_cov_2.712223_3 |  | 0.384615 | 19 | 0.0117016 | 0 | 0.1644665 |
|  | NODE_495_length_2259_cov_1.696609_1 | K06949 | 0.538462 | 5 | 0.0092817 | 0.1423759 | 0.2053679 |
|  | NODE_505_length_2160_cov_1.703313_2 |  | 0.368421 | 8 | 0.0093705 | 0.059264 | 0.004667 |
|  | NODE_570_length_1635_cov_0.354300_1 |  | 0.4251541 | 14 | 0.0160028 | 0.0260411 | 0.2614294 |
| Desulfobacterota MAG 152037 | k141_568632_length_22663_cov_15.9223_1 |  | 0.5 | 26 | 0.0058174 | 0 | 0.2486641 |
|  | k141_1047185_length_15805_cov_15.4517_10 | K02113 | 0.529412 | 20 | 0.0056116 | 0 | 0.0418443 |
|  | k141_3980423_length_15065_cov_13.4113_4 | K02601 | 0.5 | 16 | 0.0037614 | 0 | 0.0071943 |
|  | k141_1849189_length_14675_cov_16.7076_3 |  | 0.555556 | 40 | 0.0049186 | 0.0956999 | 0.0794841 |
|  | k141_1683385_length_11600_cov_21.2431_4 | K09129 | 0.529412 | 22 | 0.0041807 | 0 | 0.0674188 |
|  | k141_231468_length_10047_cov_14.2571_8 |  | 0.42507 | 56 | 0.015434 | 0 | 0.0952566 |
|  | k141_1794381_length_9459_cov_16.2750_1 |  | 0.466667 | 35 | 0.0095693 | 0 | 0.2315058 |
|  | k141_1576768_length_9406_cov_14.1930_7 | K02110 | 0.555556 | 48 | 0.0078511 | 0 | 0.0387659 |
|  | k141_699207_length_8607_cov_13.7156_5 | K02482 | 0.529412 | 83 | 0.0076115 | 0.0315385 | 0.0898286 |
|  | k141_3556753_length_7993_cov_14.0118_6 | K02004 | 0.5 | 77 | 0.0047138 | 0.1067194 | 0.1447435 |
|  | k141_3378396_length_6813_cov_14.5773_2 |  | 0.466667 | 26 | 0.0049149 | 0 | 0.1134715 |
|  | k141_1457715_length_6804_cov_13.9899_2 | K02459 | 0.452848 | 46 | 0.0074468 | 0 | 0.1186312 |
|  | k141_43637_length_5777_cov_17.4917_5 |  | 0.5 | 34 | 0.0062211 | 0 | 0.0821545 |
|  | k141_1778810_length_5586_cov_16.0000_8 |  | 0.529412 | 12 | 0.0080487 | 0 | 0.1791419 |
|  | k141_73694_length_5117_cov_12.7510_6 |  | 0.5 | 56 | 0.0052764 | 0 | 0.0513903 |
|  | k141_196843_length_5086_cov_15.3501_2 |  | 0.555556 | 16 | 0.0064364 | 0 | 0.1606928 |
|  | k141_4908399_length_4831_cov_13.4751_3 | K21071 | 0.529412 | 54 | 0.0044838 | 0 | 0.0846584 |
|  | k141_1801655_length_4808_cov_15.0467_5 | K02395 | 0.529412 | 14 | 0.0050164 | 0 | 0.2191355 |
|  | k141_1055690_length_4608_cov_12.7817_5 | K01258 | 0.5 | 62 | 0.006478 | 0 | 0.1359159 |
|  | k141_2684043_length_4025_cov_14.0111_4 | K00111 | 0.529412 | 30 | 0.0044275 | 0 | 0.1077062 |
|  | k141_146734_length_3488_cov_10.0302_2 | K01845 | 0.466667 | 74 | 0.0096508 | 0 | 0.191764 |
|  | k141_1030514_length_3060_cov_13.0058_5 | K02871 | 0.428571 | 5 | 0.0032061 | 0.1255116 | 0.0366075 |
|  | k141_1462662_length_2819_cov_15.0403_4 |  | 0.529412 | 26 | 0.004826 | 0 | 0.1342812 |
|  | k141_1026479_length_2404_cov_10.1498_5 |  | 0.5 | 15 | 0.0074884 | 0 | 0.061753 |
|  | k141_5173476_length_2192_cov_21.0965_3 | K04562 | 0.529412 | 39 | 0.0067862 | 0 | 0.0546201 |
|  | k141_3226096_length_2125_cov_8.3125_2 | K16147 | 0.4378717 | 27 | 0.0076693 | 0.1490683 | 0.1104539 |
|  | k141_5743759_length_1672_cov_11.9647_2 | K02517 | 0.555556 | 71 | 0.0168424 | 0.0314949 | 0.1090016 |
|  | k141_4130945_length_1624_cov_12.0223_1 | K02004 | 0.5 | 21 | 0.0058372 | 0 | 0.0709841 |
|  | k141_1299040_length_1539_cov_13.2082_1 |  | 0.428571 | 54 | 0.008534 | 0.1089612 | 0.1516091 |
| Desulfobacterota MAG 405075 | NODE_1_length_82503_cov_7.666598_64 | K06142 | 0.840426 | 14 | 0.003591 | 0 | 0.0288462 |
|  | NODE_1_length_82503_cov_7.666598_75 | K02849 | 0.5349077 | 51 | 0.0130283 | 0 | 0.1178097 |
|  | NODE_4_length_55705_cov_7.471197_9 | K00335 | 0.690667 | 48 | 0.0043928 | 0.1006036 | 0.1353164 |
|  | NODE_7_length_50642_cov_6.858389_23 |  | 0.538462 | 25 | 0.0032796 | 0 | 0.0528143 |
|  | NODE_7_length_50642_cov_6.858389_45 |  | 0.5732635 | 3 | 0.0024802 | 0.0581081 | 0.2440541 |
|  | NODE_10_length_44964_cov_7.097556_17 |  | 0.538462 | 8 | 0.00731 | 0 | 0.1055696 |
|  | NODE_12_length_41469_cov_6.889423_19 |  | 0.569032 | 4 | 0.0024246 | 0 | 0.0579351 |
|  | NODE_14_length_41134_cov_6.761339_19 | K17828 | 0.565957 | 19 | 0.0105317 | 0.0238994 | 0.1173996 |
|  | NODE_18_length_37848_cov_7.103073_2 | K02005 | 0.53012 | 18 | 0.0111278 | 0 | 0.0650059 |
|  | NODE_18_length_37848_cov_7.103073_8 |  | 0.65812 | 29 | 0.0082491 | 0 | 0.0408593 |
|  | NODE_44_length_23162_cov_8.507776_11 | K01470 | 0.565957 | 39 | 0.0062615 | 0 | 0.0509956 |
|  | NODE_48_length_21759_cov_7.073517_5 | K15552 | 0.777778 | 25 | 0.0145435 | 0 | 0.0829682 |
|  | NODE_62_length_19901_cov_7.632415_3 | K00219 | 0.538462 | 18 | 0.0026454 | 0 | 0.2068243 |
|  | NODE_90_length_16107_cov_6.732314_8 | K01885 | 0.538462 | 50 | 0.0107144 | 0.0332706 | 0.0410946 |
|  | NODE_96_length_15613_cov_7.541838_11 | K00176 | 0.7012175 | 7 | 0.0099353 | 0 | 0.0515979 |
|  | NODE_134_length_11243_cov_6.972416_9 |  | 0.5460527 | 4 | 0.0084402 | 0 | 0.0279387 |
|  | NODE_140_length_10757_cov_6.502013_6 | K01738 | 0.565957 | 1 | 0.0041369 | 0 | 0.0381481 |
|  | NODE_167_length_9010_cov_5.544386_1 |  | 0.565957 | 11 | 0.0118021 | 0 | 0.0410236 |
|  | NODE_175_length_8396_cov_5.893737_9 | K00176 | 0.53012 | 15 | 0.0115049 | 0 | 0.036827 |
|  | NODE_222_length_5355_cov_4.931224_3 | K02453 | 0.5291668 | 14 | 0.0093512 | 0.1987199 | 0.0711355 |
|  | NODE_235_length_4781_cov_4.392645_2 |  | 0.565957 | 7 | 0.0019107 | 0 | 0.0830986 |
|  | NODE_261_length_3760_cov_5.405105_2 |  | 0.5301935 | 18 | 0.0066989 | 0 | 0.0630277 |
| Halobacteriota MAG 203088 | NODE_1_length_83108_cov_4.054389_33 |  | 0.35014 | 6 | 0.0050444 | 0 | 0.1479369 |
|  | NODE_2_length_63211_cov_4.634587_26 | K00202 | 0.428571 | 2 | 0.0035003 | 0.0314549 | 0.5294901 |
|  | NODE_11_length_39138_cov_3.277259_10 |  | 0.3950617 | 15 | 0.0072105 | 0.1267778 | 0.3021898 |
|  | NODE_21_length_26204_cov_4.106748_7 | K03320 | 0.32 | 16 | 0.0022356 | 0 | 0.0504661 |
|  | NODE_23_length_25902_cov_3.447512_3 | K02896 | 0.35014 | 8 | 0.0103328 | 0 | 0.0119407 |
|  | NODE_27_length_22870_cov_3.743506_21 |  | 0.35014 | 11 | 0.0110091 | 0 | 0.4579929 |
|  | NODE_31_length_21126_cov_3.763837_13 |  | 0.32 | 8 | 0.0047496 | 0 | 0.0394712 |
|  | NODE_37_length_17054_cov_3.672321_2 | K03741 | 0.32 | 14 | 0.0077865 | 0.0918432 | 0.0926572 |
|  | NODE_42_length_12533_cov_3.229207_4 |  | 0.333333 | 25 | 0.0063648 | 0.0864845 | 0.2432963 |
|  | NODE_48_length_9657_cov_3.461795_7 | K00215 | 0.35014 | 13 | 0.0047047 | 0 | 0.197743 |
|  | NODE_51_length_9100_cov_2.757260_6 |  | 0.309927 | 19 | 0.0053176 | 0 | 0.067255 |
|  | NODE_72_length_4764_cov_1.794751_1 | K01575 | 0.310213 | 3 | 0.0208643 | 0.1371271 | 0.2507093 |
|  | NODE_92_length_3267_cov_1.073668_5 | K00170 | 0.428571 | 39 | 0.0107992 | 0 | 0.0531314 |
|  | NODE_96_length_3024_cov_3.503902_1 | K07787 | 0.355932 | 15 | 0.0090779 | 0.0170539 | 0.0722917 |
|  | NODE_106_length_2445_cov_1.035473_3 | K03702 | 0.384615 | 1 | 0.0131556 | 0 | 0.0236855 |
| Krumholzibacteriota MAG 2030129 | NODE_1_length_253474_cov_10.729993_10 |  | 1 | 13 | 0.0050212 | 0.0490196 | 0.1715686 |
|  | NODE_1_length_253474_cov_10.729993_24 | K03286 | 1 | 27 | 0.0034562 | 0 | 0.0545632 |
|  | NODE_2_length_248186_cov_10.076628_97 | K08591 | 0.777778 | 6 | 0.0021569 | 0 | 0.0324284 |
|  | NODE_2_length_248186_cov_10.076628_147 | K01955 | 1 | 23 | 0.0025554 | 0 | 0.1309139 |
|  | NODE_2_length_248186_cov_10.076628_152 |  | 0.836478 | 23 | 0.002852 | 0 | 0.0845471 |
|  | NODE_2_length_248186_cov_10.076628_172 | K16699 | 1 | 23 | 0.0050997 | 0 | 0.1510287 |
|  | NODE_3_length_215452_cov_10.976618_161 |  | 1 | 21 | 0.0040767 | 0 | 0.5726834 |
|  | NODE_6_length_150774_cov_10.610410_51 |  | 1 | 34 | 0.0040046 | 0 | 0.2072902 |
|  | NODE_9_length_140226_cov_11.113772_15 | K01751 | 0.803571 | 11 | 0.0029492 | 0 | 0.2308379 |
|  | NODE_9_length_140226_cov_11.113772_25 | K03650 | 0.836478 | 11 | 0.0043429 | 0 | 0.2214722 |
|  | NODE_10_length_128416_cov_9.742370_49 | K03551 | 1 | 14 | 0.0026081 | 0 | 0.0863242 |
|  | NODE_10_length_128416_cov_9.742370_76 |  | 0.836478 | 12 | 0.0041267 | 0 | 0.2084234 |
|  | NODE_12_length_116790_cov_10.980465_76 |  | 0.836478 | 22 | 0.0031928 | 0 | 0.1673004 |
|  | NODE_18_length_98125_cov_9.722136_48 | K00958 | 1 | 20 | 0.0034554 | 0.3826734 | 0.0393697 |
|  | NODE_20_length_80416_cov_10.107918_41 |  | 0.777778 | 57 | 0.00592 | 0.0153185 | 0.0843921 |
|  | NODE_22_length_73087_cov_10.817641_32 |  | 0.75 | 29 | 0.0035179 | 0 | 0.0648724 |
|  | NODE_24_length_63078_cov_10.414866_14 |  | 0.777778 | 31 | 0.0060352 | 0.0629464 | 0.1944782 |
|  | NODE_42_length_26326_cov_10.919349_1 |  | 0.836478 | 22 | 0.0058683 | 0.007746 | 0.0471832 |
|  | NODE_43_length_24639_cov_9.048612_10 |  | 1 | 12 | 0.0049157 | 0 | 0.1564193 |
|  | NODE_77_length_8378_cov_6.653415_3 | K00989 | 0.777778 | 17 | 0.00633 | 0 | 0.057659 |
| KSB1 MAG 506059 | NODE_7_length_71455_cov_4.022920_22 | K01591 | 0.53012 | 1 | 0.0051807 | 0 | 0.1472274 |
|  | NODE_38_length_44423_cov_3.271321_45 |  | 0.665772 | 4 | 0.0161298 | 0 | 0.1523759 |
|  | NODE_41_length_42017_cov_4.029876_17 |  | 0.428571 | 10 | 0.0054431 | 0 | 0.1556102 |
|  | NODE_45_length_39393_cov_4.136916_18 |  | 0.519231 | 6 | 0.0058443 | 0 | 0.0697032 |
|  | NODE_46_length_39123_cov_3.206654_11 |  | 0.491018 | 12 | 0.0075713 | 0 | 0.2153183 |
|  | NODE_46_length_39123_cov_3.206654_26 |  | 0.453642 | 13 | 0.0104835 | 0.0343721 | 0.1958985 |
|  | NODE_61_length_34296_cov_3.786142_21 |  | 0.4131113 | 14 | 0.0051078 | 0 | 0.1351875 |
|  | NODE_65_length_33299_cov_3.601981_3 |  | 0.466667 | 1 | 0.0026754 | 0 | 0.0289999 |
|  | NODE_69_length_31837_cov_3.078401_21 |  | 0.5179925 | 7 | 0.0045763 | 0 | 0.1267296 |
|  | NODE_71_length_31158_cov_3.029664_12 | K06990 | 0.533784 | 5 | 0.0066063 | 0 | 0.2014503 |
|  | NODE_72_length_30970_cov_2.914965_2 |  | 0.491018 | 9 | 0.0069935 | 0.1878378 | 0.3354247 |
|  | NODE_74_length_29685_cov_3.599973_16 | K07091 | 0.472843 | 7 | 0.0069642 | 0 | 0.1202713 |
|  | NODE_97_length_25996_cov_3.362142_15 |  | 0.466667 | 12 | 0.0132779 | 0.0939974 | 0.1575042 |
|  | NODE_109_length_23917_cov_3.491233_17 | K03390 | 0.444444 | 3 | 0.006191 | 0 | 0.0478178 |
|  | NODE_114_length_22756_cov_4.306098_1 |  | 0.555556 | 16 | 0.0058149 | 0 | 0.5616279 |
|  | NODE_125_length_20239_cov_2.844616_2 |  | 0.409091 | 3 | 0.0057403 | 0 | 0.0213148 |
|  | NODE_145_length_17735_cov_3.570846_4 | K02014 | 0.475839 | 17 | 0.0072044 | 0.0597805 | 0.1450032 |
|  | NODE_169_length_15674_cov_3.416875_6 | K12063 | 0.399285 | 16 | 0.0078012 | 0.0148092 | 0.0436612 |
|  | NODE_186_length_13945_cov_3.861119_20 |  | 0.387383 | 1 | 0.0047691 | 0.0151414 | 0.1044759 |
|  | NODE_201_length_12974_cov_3.041948_2 | K00425 | 0.491018 | 14 | 0.004298 | 0.044295 | 0.0524966 |
|  | NODE_216_length_11791_cov_1.864521_11 | K01448 | 0.5661155 | 23 | 0.0049738 | 0.0163203 | 0.1632677 |
|  | NODE_216_length_11791_cov_1.864521_12 | K02346 | 0.411765 | 33 | 0.0118762 | 0 | 0.0979831 |
|  | NODE_218_length_11636_cov_3.048793_2 | K06877 | 0.777778 | 18 | 0.0054466 | 0.0298093 | 0.0369177 |
|  | NODE_219_length_11491_cov_2.332778_10 |  | 0.409091 | 31 | 0.0069129 | 0.2751788 | 0.2946379 |
|  | NODE_230_length_10899_cov_3.485955_2 | K02014 | 0.565957 | 6 | 0.0055231 | 0 | 0.0603855 |
|  | NODE_237_length_10587_cov_3.393720_9 |  | 0.580128 | 5 | 0.0143036 | 0 | 0.0529125 |
|  | NODE_262_length_8789_cov_3.419077_2 | K02081 | 0.432352 | 1 | 0.0059516 | 0 | 0.0712102 |
|  | NODE_266_length_8656_cov_3.658352_4 | K04759 | 0.460897 | 18 | 0.0089642 | 0 | 0.0681952 |
|  | NODE_279_length_8265_cov_2.212262_7 | K03312 | 0.519393 | 6 | 0.0060785 | 0.0686072 | 0.0414094 |
|  | NODE_283_length_8112_cov_2.444431_8 |  | 0.411765 | 1 | 0.0110883 | 0 | 0.0696813 |
|  | NODE_293_length_7322_cov_3.300483_2 | K00508 | 0.519393 | 7 | 0.0049721 | 0.0845136 | 0.0478463 |
|  | NODE_310_length_6292_cov_2.541432_3 |  | 0.398374 | 7 | 0.0073668 | 0 | 0.43907 |
|  | NODE_416_length_2843_cov_3.095806_1 | K02067 | 0.399285 | 2 | 0.0109086 | 0 | 0.0179161 |
|  | NODE_585_length_535_cov_1.473799_2 |  | 0.454545 | 6 | 0.0207027 | 0 | 0.0297959 |
| Methylomirabilota MAG 51073 | k141_559976_length_54621_cov_13.2223_16 |  | 0.399285 | 16 | 0.0057883 | 0 | 0.1582237 |
|  | k141_559976_length_54621_cov_13.2223_29 |  | 0.399285 | 10 | 0.0048631 | 0 | 0.1087253 |
|  | k141_2426479_length_52056_cov_11.8866_39 | K03326 | 0.368601 | 11 | 0.003664 | 0.1185495 | 0.0454863 |
|  | k141_4928416_length_51939_cov_12.4474_22 |  | 0.399285 | 21 | 0.0038697 | 0.1379222 | 0.2527468 |
|  | k141_2089246_length_32264_cov_12.7401_27 | K03588 | 0.529412 | 9 | 0.0039932 | 0 | 0.1445956 |
|  | k141_3977365_length_29553_cov_12.9977_11 | K00058 | 0.3814135 | 2 | 0.0029563 | 0 | 0.2821816 |
|  | k141_796606_length_29340_cov_10.9510_5 |  | 0.368601 | 43 | 0.0073696 | 0.0173864 | 0.2017237 |
|  | k141_1586185_length_29206_cov_11.0151_13 | K18889 | 0.366456 | 41 | 0.0045648 | 0 | 0.3448538 |
|  | k141_4166638_length_26776_cov_11.1373_15 | K01974 | 0.399285 | 15 | 0.0057857 | 0 | 0.563476 |
|  | k141_3237653_length_25136_cov_11.5218_8 | K03979 | 0.399285 | 9 | 0.0040729 | 0 | 0.1387861 |
|  | k141_341363_length_24544_cov_11.3113_1 | K01207 | 0.399285 | 5 | 0.0052602 | 0 | 0.2519871 |
|  | k141_870669_length_21237_cov_11.1642_2 |  | 0.399285 | 23 | 0.0073112 | 0 | 0.2446925 |
|  | k141_3380771_length_20355_cov_10.2141_16 | K03642 | 0.399285 | 21 | 0.0088665 | 0.0355357 | 0.1569494 |
|  | k141_1529782_length_17060_cov_9.1928_16 | K01703 | 0.399285 | 10 | 0.0045826 | 0.0651208 | 0.0489064 |
|  | k141_4584448_length_16201_cov_10.7838_6 | K07714 | 0.368601 | 13 | 0.0053464 | 0.035533 | 0.054498 |
|  | k141_474108_length_16177_cov_9.5879_9 | K00145 | 0.399285 | 28 | 0.0063698 | 0 | 0.1697989 |
|  | k141_1202118_length_16173_cov_9.7712_21 | K07082 | 0.399285 | 12 | 0.0051346 | 0 | 0.0940053 |
|  | k141_2062083_length_15878_cov_11.4127_3 | K09015 | 0.399285 | 27 | 0.005239 | 0 | 0.1664351 |
|  | k141_2630051_length_15452_cov_10.1611_8 | K13628 | 0.529412 | 4 | 0.0072783 | 0 | 0.0297132 |
|  | k141_311592_length_14389_cov_11.1684_9 | K02027 | 0.555556 | 7 | 0.0047164 | 0.0927853 | 0.1019799 |
|  | k141_4400527_length_13817_cov_13.5540_10 |  | 0.368601 | 17 | 0.0037131 | 0.3716216 | 0.2136995 |
|  | k141_2681535_length_12614_cov_14.2283_1 | K19225 | 0.5 | 20 | 0.0044823 | 0 | 0.1896284 |
|  | k141_3620884_length_9292_cov_12.0258_3 | K02671 | 0.368601 | 16 | 0.0052744 | 0 | 0.040537 |
|  | k141_1514645_length_9033_cov_9.2736_7 | K14126 | 0.368601 | 11 | 0.0061905 | 0.0108457 | 0.0804641 |
|  | k141_3313134_length_8751_cov_11.8092_1 |  | 0.399285 | 5 | 0.0050898 | 0 | 0.1300461 |
|  | k141_33602_length_8442_cov_10.6404_9 |  | 0.399285 | 11 | 0.0103002 | 0 | 0.0689766 |
|  | k141_538975_length_8101_cov_8.0000_9 |  | 0.368601 | 24 | 0.0065573 | 0.0294602 | 0.0445361 |
|  | k141_2326132_length_7947_cov_11.1925_3 | K02460 | 0.529412 | 29 | 0.0062282 | 0 | 0.0855949 |
|  | k141_2780833_length_7301_cov_9.0022_3 | K01999 | 0.368601 | 13 | 0.0039473 | 0.130053 | 0.1642187 |
|  | k141_2654115_length_7263_cov_10.4183_1 | K00041 | 0.399285 | 28 | 0.0045214 | 0 | 0.3880869 |
|  | k141_2091750_length_6067_cov_10.9879_3 |  | 0.529412 | 2 | 0.0032314 | 0 | 0.4065805 |
|  | k141_1403093_length_5869_cov_9.4859_5 | K21393 | 0.4496425 | 4 | 0.0029946 | 0 | 0.0359998 |
|  | k141_1198952_length_5274_cov_12.1638_2 | K03457 | 0.368601 | 21 | 0.0034671 | 0.0186288 | 0.1583559 |
|  | k141_3372116_length_4884_cov_9.5906_1 |  | 0.399285 | 9 | 0.0080855 | 0 | 0.0468567 |
|  | k141_5499612_length_4850_cov_10.4150_2 | K02027 | 0.399285 | 17 | 0.0037369 | 0 | 0.0283725 |
|  | k141_4226485_length_4654_cov_10.6076_5 | K01524 | 0.555556 | 7 | 0.0049764 | 0 | 0.3606492 |
|  | k141_1484461_length_3939_cov_10.9613_1 |  | 0.368601 | 5 | 0.0101621 | 0 | 0.2272794 |
|  | k141_586634_length_3403_cov_9.2379_2 |  | 0.368601 | 20 | 0.0052009 | 0 | 0.347902 |
|  | k141_4018098_length_2676_cov_10.6568_3 |  | 0.368601 | 8 | 0.0062974 | 0.0133831 | 0.0913814 |
| Nitrospirota MAG 51092 | NODE_2_length_96825_cov_5.568735_13 |  | 0.35014 | 7 | 0.0099627 | 0.0120935 | 0.1096237 |
|  | NODE_3_length_79520_cov_6.310323_17 |  | 0.399285 | 22 | 0.0063448 | 0 | 0.2476062 |
|  | NODE_4_length_74377_cov_6.450673_32 |  | 0.35014 | 15 | 0.0237217 | 0 | 0.1836735 |
|  | NODE_7_length_62210_cov_6.018975_51 | K02004 | 0.35014 | 9 | 0.0041994 | 0 | 0.0552889 |
|  | NODE_9_length_52427_cov_5.902004_4 | K00606 | 0.35014 | 4 | 0.0057785 | 0 | 0.4922395 |
|  | NODE_9_length_52427_cov_5.902004_26 | K07315 | 0.399285 | 18 | 0.0059014 | 0 | 0.1215441 |
|  | NODE_9_length_52427_cov_5.902004_45 |  | 0.35014 | 18 | 0.0113243 | 0.1075865 | 0.1350799 |
|  | NODE_9_length_52427_cov_5.902004_48 | K06911 | 0.555556 | 11 | 0.0040136 | 0 | 0.2174914 |
|  | NODE_29_length_28738_cov_6.124315_17 | K03734 | 0.399285 | 6 | 0.0067792 | 0 | 0.1536795 |
|  | NODE_34_length_27756_cov_5.271939_6 | K02574 | 0.399285 | 14 | 0.0048302 | 0 | 0.2609518 |
|  | NODE_40_length_22902_cov_5.561402_5 |  | 0.555556 | 15 | 0.0225835 | 0.0297631 | 0.2883953 |
|  | NODE_45_length_20311_cov_6.634674_13 | K03672 | 0.399285 | 3 | 0.0015981 | 0 | 0.0309091 |
|  | NODE_53_length_18520_cov_5.761373_9 |  | 0.35014 | 10 | 0.0047732 | 0 | 0.2317537 |
|  | NODE_58_length_17145_cov_7.424889_6 | K00991 | 0.555556 | 4 | 0.0040946 | 0 | 0.4195894 |
|  | NODE_71_length_13130_cov_4.399372_14 |  | 0.555556 | 4 | 0.0244787 | 0 | 0.2767442 |
|  | NODE_72_length_13048_cov_6.069766_6 | K00817 | 0.399285 | 15 | 0.0049724 | 0.1906898 | 0.5741882 |
|  | NODE_77_length_11089_cov_5.935071_14 | K04754 | 0.399285 | 14 | 0.0058665 | 0.030315 | 0.1036002 |
|  | NODE_83_length_8907_cov_4.779502_8 |  | 0.35014 | 1 | 0.0062115 | 0 | 0.0992169 |
|  | NODE_121_length_2657_cov_3.435271_4 | K06180 | 0.399285 | 23 | 0.0127727 | 0 | 0.3406153 |
| Pseudomonadota MAG 0532 | NODE_4_length_30681_cov_4.815220_13 |  | 0.399285 | 45 | 0.012305 | 0 | 0.4368549 |
|  | NODE_5_length_27589_cov_4.492640_14 | K03087 | 0.35014 | 51 | 0.0097003 | 0 | 0.0812084 |
|  | NODE_6_length_24240_cov_4.900890_22 |  | 0.555556 | 24 | 0.0119532 | 0.133792 | 0.1083007 |
|  | NODE_7_length_23986_cov_4.937597_8 | K15011 | 0.606061 | 99 | 0.0144716 | 0.1384763 | 0.133126 |
|  | NODE_8_length_22815_cov_4.815375_3 | K03638 | 0.332511 | 27 | 0.0073534 | 0 | 0.1727757 |
|  | NODE_11_length_19914_cov_5.416494_11 | K00647 | 0.35014 | 50 | 0.0069513 | 0 | 0.0418278 |
|  | NODE_20_length_17193_cov_4.437770_2 |  | 0.35014 | 33 | 0.0084752 | 0 | 0.0902952 |
|  | NODE_21_length_16976_cov_3.879105_16 | K07738 | 0.555556 | 26 | 0.0063038 | 0 | 0.024458 |
|  | NODE_29_length_13860_cov_4.678952_13 | K03584 | 0.399285 | 29 | 0.0099397 | 0 | 0.1219949 |
|  | NODE_32_length_13430_cov_5.107766_13 | K00855 | 0.399285 | 29 | 0.006524 | 0 | 0.0117674 |
|  | NODE_50_length_11150_cov_3.986363_6 | K03308 | 0.529412 | 82 | 0.0170176 | 0.0348974 | 0.0583221 |
|  | NODE_51_length_11141_cov_5.069234_5 |  | 0.555556 | 28 | 0.0084785 | 0 | 0.2176086 |
|  | NODE_60_length_10451_cov_4.336996_12 | K01953 | 0.333333 | 17 | 0.0107921 | 0.0112334 | 0.0865402 |
|  | NODE_62_length_10131_cov_4.556296_2 |  | 0.555556 | 15 | 0.0103946 | 0 | 0.0688825 |
|  | NODE_64_length_9773_cov_3.481126_4 | K22225 | 0.399285 | 72 | 0.0150733 | 0 | 0.2585927 |
|  | NODE_68_length_9556_cov_3.151266_7 | K22616 | 0.370525 | 60 | 0.0131915 | 0 | 0.2415221 |
|  | NODE_79_length_8611_cov_5.041481_6 |  | 0.399285 | 20 | 0.0053697 | 0 | 0.102942 |
|  | NODE_97_length_7438_cov_5.255128_1 | K01775 | 0.399285 | 5 | 0.008684 | 0.1031349 | 0.0397806 |
|  | NODE_97_length_7438_cov_5.255128_6 | K03106 | 0.368601 | 63 | 0.0099609 | 0 | 0.0443551 |
|  | NODE_112_length_6368_cov_4.173104_2 |  | 0.399285 | 107 | 0.0112963 | 0.5514872 | 0.1472889 |
|  | NODE_139_length_5659_cov_3.748119_7 | K07659 | 0.3829033 | 40 | 0.0177643 | 0 | 0.1288412 |
|  | NODE_142_length_5634_cov_3.770380_4 |  | 0.399285 | 30 | 0.0099811 | 0 | 0.08199 |
|  | NODE_143_length_5630_cov_4.211417_3 |  | 0.399285 | 37 | 0.0139119 | 0.3275748 | 0.4355664 |
|  | NODE_153_length_5333_cov_4.319064_4 |  | 0.3970043 | 10 | 0.0260001 | 0 | 0.6237143 |
|  | NODE_153_length_5333_cov_4.319064_5 | K00950 | 0.529412 | 23 | 0.0128867 | 0 | 0.0564503 |
|  | NODE_165_length_4857_cov_5.410251_6 | K03978 | 0.555556 | 16 | 0.0152564 | 0 | 0.1304627 |
|  | NODE_177_length_4472_cov_5.127418_1 | K23916 | 0.399285 | 59 | 0.0106973 | 0 | 0.0474503 |
|  | NODE_203_length_4033_cov_3.277300_2 | K00703 | 0.35014 | 94 | 0.0141723 | 0.132967 | 0.1611709 |
|  | NODE_204_length_4023_cov_1.158895_6 |  | 0.370525 | 16 | 0.0182316 | 0 | 0.0602099 |
|  | NODE_220_length_3727_cov_3.188493_1 |  | 0.35014 | 56 | 0.0135176 | 0 | 0.0665354 |
|  | NODE_221_length_3723_cov_3.577345_2 |  | 0.399285 | 18 | 0.0110658 | 0 | 0.0997766 |
|  | NODE_263_length_3043_cov_3.384019_3 |  | 0.368601 | 54 | 0.0130906 | 0.0359639 | 0.1104865 |
|  | NODE_310_length_2615_cov_3.484240_2 | K09889 | 0.35014 | 33 | 0.0106893 | 0 | 0.2968304 |
|  | NODE_368_length_2077_cov_3.648500_1 |  | 0.35014 | 26 | 0.0154567 | 0 | 0.1085647 |
|  | NODE_375_length_2020_cov_3.039629_3 | K03593 | 0.347953 | 2 | 0.0095602 | 0.0626728 | 0.0044766 |
|  | NODE_404_length_1631_cov_2.736165_1 |  | 0.31635 | 59 | 0.0122395 | 0 | 0.1455385 |
|  | NODE_410_length_1525_cov_2.035221_2 |  | 0.35014 | 22 | 0.0292709 | 0.076298 | 0.7766043 |
| Pseudomonadota MAG 101530 | k141_1123624_length_36189_cov_15.4380_5 | K01262 | 0.399285 | 26 | 0.0069446 | 0 | 0.1140899 |
|  | k141_1298643_length_26895_cov_14.1902_17 | K07100 | 0.555556 | 17 | 0.008413 | 0.1520848 | 0.1083324 |
|  | k141_4369372_length_25107_cov_15.9634_6 | K06131 | 0.384615 | 14 | 0.0074254 | 0 | 0.0669701 |
|  | k141_3357589_length_21230_cov_16.8577_13 | K00362 | 0.402778 | 49 | 0.0060167 | 0.229601 | 0.0493042 |
|  | k141_1163615_length_19909_cov_14.0787_11 |  | 0.5 | 30 | 0.0093343 | 0 | 0.1078249 |
|  | k141_6170492_length_19096_cov_13.8771_17 | K03499 | 0.4351853 | 46 | 0.0069602 | 0 | 0.0206662 |
|  | k141_3017975_length_18823_cov_14.9551_11 |  | 0.368601 | 4 | 0.0046744 | 0 | 0.0744439 |
|  | k141_2945133_length_15426_cov_11.5237_7 |  | 0.368601 | 25 | 0.0058031 | 0 | 0.0853369 |
|  | k141_6286294_length_11748_cov_16.1548_6 |  | 0.555556 | 12 | 0.0073347 | 0 | 0.0383782 |
|  | k141_5797102_length_11397_cov_14.9035_6 |  | 0.368601 | 13 | 0.0113947 | 0 | 0.0864875 |
|  | k141_2827426_length_10739_cov_12.0937_5 |  | 0.368601 | 2 | 0.0074207 | 0 | 0.0555778 |
|  | k141_2968032_length_10082_cov_14.5690_10 |  | 0.368601 | 8 | 0.0057467 | 0 | 0.2527395 |
|  | k141_2878631_length_9440_cov_15.9772_11 | K07213 | 0.368601 | 7 | 0.0056625 | 0 | 0.1746285 |
|  | k141_176564_length_9014_cov_9.3491_5 | K11178 | 0.5 | 3 | 0.0056581 | 0 | 0.205204 |
|  | k141_3517428_length_8737_cov_12.1427_5 | K00681 | 0.368601 | 7 | 0.0085707 | 0 | 0.1142335 |
|  | k141_807137_length_7830_cov_16.5033_6 | K14415 | 0.466667 | 24 | 0.0077237 | 0 | 0.0949587 |
|  | k141_5843294_length_7696_cov_15.0000_2 | K00351 | 0.399285 | 4 | 0.0048606 | 0 | 0.0410885 |
|  | k141_2690237_length_6215_cov_12.9174_4 | K04042 | 0.399285 | 21 | 0.0061153 | 0 | 0.1097904 |
|  | k141_1726210_length_5621_cov_26.1631_5 | K07712 | 0.506958 | 17 | 0.0075295 | 0 | 0.0278577 |
|  | k141_911316_length_5614_cov_12.8856_5 | K03545 | 0.368601 | 27 | 0.00601 | 0.031789 | 0.0928004 |
|  | k141_275475_length_3567_cov_12.0628_4 | K02302 | 0.529412 | 11 | 0.0088859 | 0.0485294 | 0.0371107 |
|  | k141_3886249_length_3019_cov_9.8297_3 | K19304 | 0.5 | 11 | 0.0076646 | 0.0511879 | 0.1022675 |
|  | k141_1351053_length_2627_cov_9.6380_2 |  | 0.368601 | 15 | 0.0057752 | 0 | 0.154441 |
|  | k141_1820055_length_2347_cov_14.9238_1 | K02119 | 0.414586 | 10 | 0.0060022 | 0 | 0.1539235 |
|  | k141_2235706_length_2260_cov_12.7702_1 |  | 0.375 | 33 | 0.0088964 | 0 | 0.0297051 |
|  | k141_2769712_length_2161_cov_10.0000_1 |  | 0.466667 | 20 | 0.0093762 | 0 | 0.0931543 |
|  | k141_786884_length_2004_cov_6.9275_1 |  | 0.368601 | 69 | 0.0153019 | 0.0688086 | 0.0418737 |
|  | k141_161485_length_1844_cov_10.9237_3 | K00574 | 0.396601 | 3 | 0.0081194 | 0.0515587 | 0.0951789 |
|  | k141_5332773_length_1583_cov_7.5541_2 |  | 0.433628 | 6 | 0.0098171 | 0.0736617 | 0.0954156 |
| Pseudomonadota MAG 152079 | k141_5808927_length_45009_cov_10.9801_11 | K04564 | 0.289826 | 7 | 0.005615 | 0 | 0.0833478 |
|  | k141_3995293_length_28476_cov_11.3452_14 |  | 0.399285 | 10 | 0.0083241 | 0 | 0.2459985 |
|  | k141_397653_length_24930_cov_11.5185_17 | K00525 | 0.368601 | 100 | 0.0092947 | 0.1710347 | 0.0639731 |
|  | k141_2276636_length_20709_cov_11.2339_1 | K03070 | 0.35014 | 57 | 0.0087946 | 0.0948511 | 0.0647365 |
|  | k141_5617125_length_16723_cov_11.3817_7 | K01817 | 0.399285 | 19 | 0.0078057 | 0 | 0.4448656 |
|  | k141_2240388_length_12915_cov_12.3646_14 |  | 0.304348 | 10 | 0.0058503 | 0 | 0.0849182 |
|  | k141_1114009_length_12163_cov_11.0500_6 |  | 0.278477 | 8 | 0.0097411 | 0 | 0.2735588 |
|  | k141_3474027_length_8054_cov_10.4918_4 | K00342 | 0.2917775 | 37 | 0.011004 | 0 | 0.0806949 |
|  | k141_1629928_length_7163_cov_13.0000_1 | K02968 | 0.399285 | 2 | 0.0100096 | 0 | 0.1263724 |
|  | k141_1260735_length_4226_cov_8.0698_5 |  | 0.3062951 | 7 | 0.0153397 | 0 | 0.2130291 |
|  | k141_2176049_length_4002_cov_13.8415_1 | K00956 | 0.368601 | 28 | 0.0093249 | 0.0840199 | 0.0906348 |
|  | k141_5886775_length_3587_cov_11.1582_3 | K01895 | 0.344994 | 51 | 0.0070595 | 0 | 0.0626434 |
|  | k141_223403_length_3205_cov_12.0000_3 | K02199 | 0.399285 | 4 | 0.0086551 | 0 | 0.272482 |
|  | k141_4677169_length_2082_cov_7.0000_2 |  | 0.2917775 | 6 | 0.0218214 | 0.2546479 | 0.3589828 |
|  | k141_1993772_length_1646_cov_8.0000_2 |  | 0.368601 | 9 | 0.0055328 | 0 | 0.0545882 |
| Pseudomonadota MAG 203040 | NODE_1_length_179414_cov_6.373961_36 | K03702 | 0.384615 | 11 | 0.0026762 | 0 | 0.0727032 |
|  | NODE_2_length_171074_cov_7.322158_96 | K01259 | 0.305235 | 15 | 0.0058213 | 0 | 0.4246474 |
|  | NODE_2_length_171074_cov_7.322158_105 |  | 0.384615 | 17 | 0.0062693 | 0 | 0.2801043 |
|  | NODE_3_length_154099_cov_6.840977_125 | K01673 | 0.384615 | 19 | 0.0093219 | 0 | 0.3462991 |
|  | NODE_4_length_129719_cov_6.937626_93 | K01937 | 0.399285 | 6 | 0.0032945 | 0 | 0.0413076 |
|  | NODE_7_length_94617_cov_7.138767_11 | K00287 | 0.35014 | 7 | 0.0078554 | 0.0326923 | 0.3563462 |
|  | NODE_8_length_94384_cov_7.426416_16 | K00428 | 0.36036 | 24 | 0.0054424 | 0.0347961 | 0.2375355 |
|  | NODE_10_length_91911_cov_6.542222_85 |  | 0.35014 | 4 | 0.0185662 | 0 | 0.313892 |
|  | NODE_12_length_79994_cov_6.753694_18 | K02003 | 0.555556 | 9 | 0.0072538 | 0 | 0.0825688 |
|  | NODE_12_length_79994_cov_6.753694_20 | K07305 | 0.35014 | 17 | 0.0090944 | 0 | 0.1512103 |
|  | NODE_12_length_79994_cov_6.753694_54 | K02005 | 0.36036 | 32 | 0.009236 | 0.0894053 | 0.1214268 |
|  | NODE_13_length_73840_cov_6.773637_58 |  | 0.2983035 | 4 | 0.0054185 | 0 | 0.2121831 |
|  | NODE_20_length_54529_cov_6.990763_6 |  | 0.2968875 | 8 | 0.0042116 | 0 | 0.1237435 |
|  | NODE_21_length_53992_cov_6.486581_12 | K00381 | 0.35014 | 12 | 0.0048397 | 0 | 0.0939849 |
|  | NODE_24_length_52771_cov_7.220143_27 |  | 0.35014 | 5 | 0.0032357 | 0 | 0.0309754 |
|  | NODE_31_length_44289_cov_7.502669_2 | K02115 | 0.2917775 | 13 | 0.0047676 | 0 | 0.0733532 |
|  | NODE_34_length_41939_cov_7.251636_10 |  | 0.294778 | 10 | 0.0175666 | 0 | 0.3468568 |
|  | NODE_36_length_41278_cov_7.336683_37 |  | 0.318182 | 2 | 0.0052009 | 0 | 0.1397516 |
|  | NODE_42_length_35095_cov_6.421498_29 | K02477 | 0.368601 | 17 | 0.0117602 | 0 | 0.0431345 |
|  | NODE_48_length_33033_cov_6.543179_20 |  | 0.399285 | 6 | 0.0058154 | 0 | 0.2224617 |
|  | NODE_51_length_30251_cov_6.244921_17 |  | 0.399285 | 10 | 0.0053198 | 0 | 0.0582693 |
|  | NODE_58_length_26633_cov_6.173369_10 | K14063 | 0.35014 | 32 | 0.0142979 | 0 | 0.2826584 |
|  | NODE_61_length_25722_cov_7.625931_23 |  | 0.35014 | 10 | 0.0067652 | 0 | 0.9277407 |
|  | NODE_102_length_11483_cov_4.673330_13 |  | 0.35014 | 29 | 0.011246 | 0 | 0.0253752 |
|  | NODE_103_length_11344_cov_5.229342_4 | K15580 | 0.35014 | 28 | 0.0076818 | 0.154113 | 0.2896052 |
|  | NODE_118_length_9510_cov_6.983886_2 |  | 0.384615 | 49 | 0.0105426 | 0 | 0.188992 |
|  | NODE_119_length_9304_cov_4.819009_1 | K02014 | 0.2845103 | 19 | 0.0076237 | 0.0547953 | 0.4048824 |
|  | NODE_119_length_9304_cov_4.819009_6 | K00428 | 0.399285 | 38 | 0.0114034 | 0.034428 | 0.1277654 |
|  | NODE_150_length_6300_cov_5.420215_6 |  | 0.2913488 | 19 | 0.0102769 | 0.0702745 | 0.1493758 |
|  | NODE_152_length_6158_cov_6.570301_10 |  | 0.384615 | 9 | 0.0095157 | 0.0105413 | 0.2249064 |
|  | NODE_164_length_4462_cov_3.574002_1 |  | 0.399285 | 49 | 0.0088403 | 0 | 0.1599887 |
| Pseudomonadota MAG 4050181 | NODE_3_length_85099_cov_6.404364_22 | K01468 | 0.35014 | 27 | 0.0085713 | 0.2004483 | 0.2572071 |
|  | NODE_3_length_85099_cov_6.404364_82 | K04654 | 0.35014 | 49 | 0.0070056 | 0 | 0.1396043 |
|  | NODE_11_length_58689_cov_4.853083_8 | K10823 | 0.32 | 2 | 0.0046858 | 0 | 0.1462146 |
|  | NODE_11_length_58689_cov_4.853083_26 | K02884 | 0.35014 | 14 | 0.0052376 | 0 | 0.0993186 |
|  | NODE_21_length_45488_cov_6.057695_1 |  | 0.35014 | 44 | 0.0155528 | 0 | 0.0369457 |
|  | NODE_21_length_45488_cov_6.057695_5 | K00389 | 0.35014 | 10 | 0.0103886 | 0 | 0.1408832 |
|  | NODE_25_length_40624_cov_4.971317_7 |  | 0.384615 | 2 | 0.0038781 | 0 | 0.0523473 |
|  | NODE_35_length_34060_cov_4.245983_25 |  | 0.399285 | 26 | 0.0082598 | 0 | 0.1579849 |
|  | NODE_36_length_33889_cov_4.404004_1 |  | 0.3847141 | 49 | 0.005647 | 0.2889811 | 0.365359 |
|  | NODE_36_length_33889_cov_4.404004_13 |  | 0.344994 | 5 | 0.0111666 | 0 | 0.201005 |
|  | NODE_36_length_33889_cov_4.404004_30 |  | 0.32 | 8 | 0.0067594 | 0.1251163 | 0.0279856 |
|  | NODE_37_length_33486_cov_6.834805_8 | K14393 | 0.35014 | 69 | 0.0070404 | 0 | 0.04159 |
|  | NODE_38_length_30515_cov_4.436672_10 | K11690 | 0.4177885 | 6 | 0.0064757 | 0.5713141 | 0.1124119 |
|  | NODE_45_length_28333_cov_4.609782_21 |  | 0.35014 | 4 | 0.0118162 | 0 | 0.1958904 |
|  | NODE_47_length_27579_cov_4.918179_19 | K00253 | 0.399285 | 14 | 0.0060533 | 0.1489784 | 0.0621633 |
|  | NODE_49_length_26483_cov_5.212679_13 | K09004 | 0.321974 | 17 | 0.0114456 | 0.2301784 | 0.313349 |
|  | NODE_60_length_22519_cov_3.732320_3 | K14136 | 0.35014 | 22 | 0.0079929 | 0.0257844 | 0.1085967 |
|  | NODE_60_length_22519_cov_3.732320_13 | K13993 | 0.35014 | 5 | 0.0106188 | 0 | 0.1510493 |
|  | NODE_66_length_21012_cov_4.333413_10 | K01420 | 0.35014 | 17 | 0.0058939 | 0 | 0.1757638 |
|  | NODE_70_length_19104_cov_4.324223_18 | K01662 | 0.2649731 | 26 | 0.0046816 | 0.0504594 | 0.1908305 |
|  | NODE_80_length_17278_cov_4.829312_18 |  | 0.32302 | 16 | 0.010504 | 0 | 0.2295214 |
|  | NODE_80_length_17278_cov_4.829312_20 |  | 0.375 | 3 | 0.0055062 | 0 | 0.1683986 |
|  | NODE_90_length_15925_cov_4.373801_20 |  | 0.35014 | 10 | 0.0087011 | 0 | 0.0428926 |
|  | NODE_94_length_14218_cov_4.315090_10 | K01887 | 0.35014 | 39 | 0.0088356 | 0.1353215 | 0.2015935 |
|  | NODE_117_length_9700_cov_3.745713_9 | K02053 | 0.35014 | 24 | 0.0085263 | 0 | 0.0512852 |
|  | NODE_126_length_8561_cov_2.987269_6 |  | 0.299052 | 33 | 0.0143799 | 0.0355222 | 0.114328 |
|  | NODE_147_length_5592_cov_3.514778_5 | K09969 | 0.399285 | 14 | 0.0064047 | 0 | 0.0289586 |
|  | NODE_163_length_4043_cov_4.260464_1 | K07080 | 0.367246 | 1 | 0.0041439 | 0.1936786 | 0.0382967 |
|  | NODE_205_length_1769_cov_1.413712_1 |  | 0.32 | 13 | 0.021706 | 0.0396001 | 0.0663453 |
|  | NODE_389_length_551_cov_1.118143_2 |  | 0.3405525 | 3 | 0.0134524 | 0 | 0.2700189 |
